# Investigations of thiol-modified phenol derivatives for the use in thiol–ene photopolymerizations

**DOI:** 10.3762/bjoc.10.180

**Published:** 2014-07-29

**Authors:** Sebastian Reinelt, Monir Tabatabai, Urs Karl Fischer, Norbert Moszner, Andreas Utterodt, Helmut Ritter

**Affiliations:** 1 Heinrich-Heine-University Düsseldorf, Institute of Organic Chemistry and Macromolecular Chemistry, Department of Preparative Polymer Chemistry, Universitätsstraße 1, 40225 Düsseldorf, Germany; 2 Ivoclar Vivadent AG, Bendererstrasse 2, 9494 Schaan, Principality of Liechtenstein; 3 Heraeus Kulzer GmbH, Philipp-Reis-Straße 8, 61273 Wehrheim, Germany

**Keywords:** composites, crosslinking, dental polymers, high performance polymers, photopolymerization

## Abstract

Thiol–ene photopolymerizations gain a growing interest in academic research. Coatings and dental restoratives are interesting applications for thiol–ene photopolymerizations due to their unique features. In most studies the relative flexible and hydrophilic ester derivative, namely pentaerythritoltetra(3-mercaptopropionate) (PETMP), is investigated as the thiol component. Thus, in the present study we are encouraged to investigate the performance of more hydrophobic ester-free thiol-modified bis- and trisphenol derivatives in thiol–ene photopolymerizations. For this, six different thiol-modified bis- and trisphenol derivatives exhibiting four to six thiol groups are synthesized via the radical addition of thioacetic acid to suitable allyl-modified precursors and subsequent hydrolysis. Compared to PETMP better flexural strength and modulus of elasticity are achievable in thiol–ene photopolymerizations employing 1,3,5-triallyl-1,3,5-triazine-2,4,6-trione (TATATO) as the ene derivative. Especially, after storage in water, the flexural strength and modulus of elasticity is twice as high compared to the PETMP reference system.

## Introduction

Restorative composites consist of a polymerizable organic matrix (10–60 wt %) and inorganic fillers (40–90 wt %) [1]. Up to date, 2,2-bis(4-(2-hydroxy-3-methacryloxyprop-1-oxy)phenyl)propane (Bis-GMA), 1,6-bis(methacryloxy-2-ethoxycarbonylamino)-(mixture of 2,2,4 and 2,4,4)-trimethylhexane (UDMA) and triethylene glycol dimethacrylate (TEGDMA) are still the dominating monomers of the organic matrix used in the formulation of dental composites [1–4].

The shrinkage stress and the leachability of unreacted monomers due to an insufficient double bond conversion are two of the main drawbacks of dimethacrylate based dental resins and are still a driving force for dental researches [2]. Many efforts have been undertaken in the last decades aiming to face and overcome these limitations [1,4–8]. For instance the design of tailored (meth)acrylates based on calix[4]arenes [9–10] or tricyclodecane [11–12] backbones and use of ring-opening monomers as cyclopropane derivatives [13–14], siloranes [15] or spiroorthocarbonates [16] are worth to be mentioned.

Since Sharpless et al. published their concept of click chemistry in 2001, these reactions gained growing impact on the design of new materials [17]. Besides the alkyne–azide reaction, especially thiol–ene reactions were studied extensively [18–21]. Bowman and coworkers first investigated thiol–ene photopolymerizations as promising approach towards novel, low-shrinkage dental restoratives [22–23]. In contrast to the chain-growth mechanism of dimethylacrylate-based systems thiol–ene polymerizations proceed via a step-growth mechanism [24] which offers the advantage of a delayed gel point and thus, a reduced shrinkage stress [22,25]. These preliminary studies were an offense for further investigations in the field of thiol-based materials covering the topics of ternary thiol–ene-methacrylate system [26–28], adaptable thiol–ene networks [29], thiol–yne photopolymerizations [30–32], and thiol–norbornene materials [33] only to mention a few of them. However, the design of new monomers – both ene and thiol monomers – for thiol–ene polymerization is a sustained necessity in order to improve the mechanical properties of the resulting networks [34]. So far most studies focused on the optimization of the ene monomer while PETMP (pentaerythritol tetra(3-mercaptopropionate)) was constantly utilized as the thiol component.

The influence of water sorption of cured dental composites on the mechanical properties have been investigated and discussed extensively in several studies dealing with dimethacrylate based composites. The use of hydrophilic monomers leads to a higher water uptake which induces reduced strength, stiffness and wear-resistance and may cause erosion of the filler or of the unreacted monomer [35–38]. For studies focusing on thiol–ene photopolymerizations for dental restoratives, the mechanical properties of the cured composites are always determined under ambient conditions. However, the mainly investigated PETMP monomer has a partial hydrophilic character due to its four ester linkages and thus, a significant influence of water sorption on the mechanical properties should be expected.

The limited availability of rigid thiol monomers is the initial point for this study. In analogy to Bis-GMA which is a well-established monomer for dental materials exhibiting good mechanical properties based on its rigid structure, we wish to present the synthesis of various multifunctional, hydrophobic thiol monomers all having in common a rigid phenol backbone and at least four primary aliphatic groups as well as the fact that they are ester-free liquids. Their behavior in thiol–ene photopolymerizations regarding the conversion and mechanical properties before and after water storage is investigated and compared to the widely used PETMP. Thereby, the well-established, commercially available triallyl-1,3,5-triazine-2,4,6-trione (TATATO) is utilized as the ene monomer.

## Results and Discussion

**Scheme 1:**
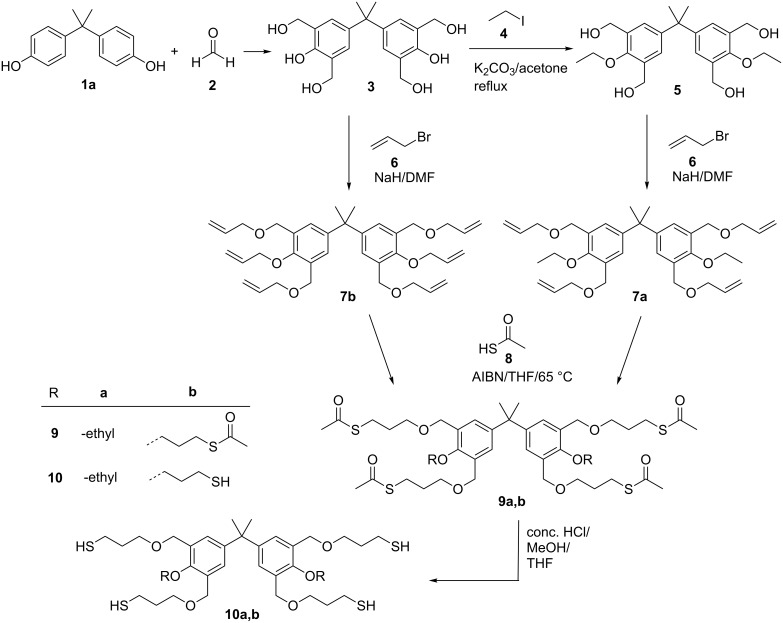
Synthetic route for the preparation of the thiol-functionalized bisphenols **10a** and **10b**.

**Scheme 2:**
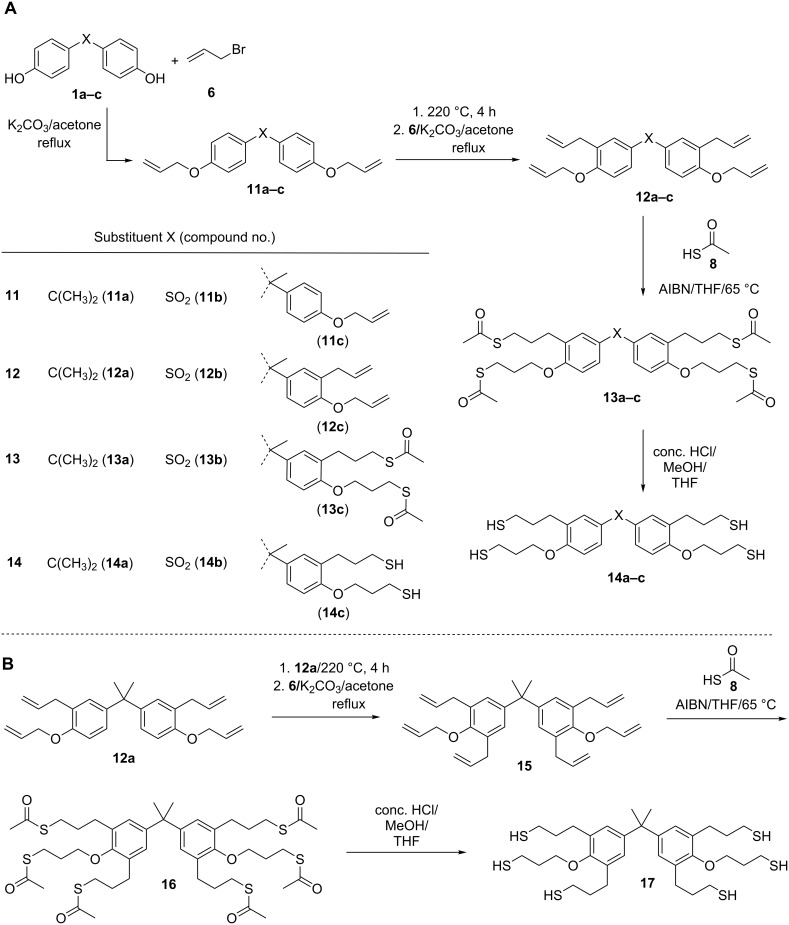
Synthetic route for the preparation of thiol-functionalized phenol derivatives **14a–c** and **17** using the Claisen rearrangement and the radical addition of thioacetic acid as key steps.

**Synthesis and characterization of the thiol-modified bis- and trisphenol derivatives.** Most synthetic routes for the preparation of thiols make use of alkyl halogenide or allyl derivatives as suitable precursors which are subsequently converted into the corresponding thiols [39]. In the present study, the synthesis of the phenol-based thiols (**10a,b**, **14a–c**, **17**) was accomplished by the radical addition of thioacetic acid to suitable allyl-modified precursors as depicted in Scheme 1 and Scheme 2. For the synthesis of **10a** and **10b** the well-established hydroxymethylation of bisphenol A was the starting point of the reaction sequence and yielded compound **3**. The quantitative etherification of the phenolic hydroxy groups with potassium carbonate and ethyl iodide yielded the corresponding ether derivative **5**. Subsequently the remaining hydroxy groups of **3** and **5** were o-alkylated in the presence of a strong base such as sodium hydride and allyl bromide (**6**), which resulted in the formation of the tetra-allyl (**7a**) and hexa-allyl (**7b**) derivatives (Scheme 1).

**Figure 1:**
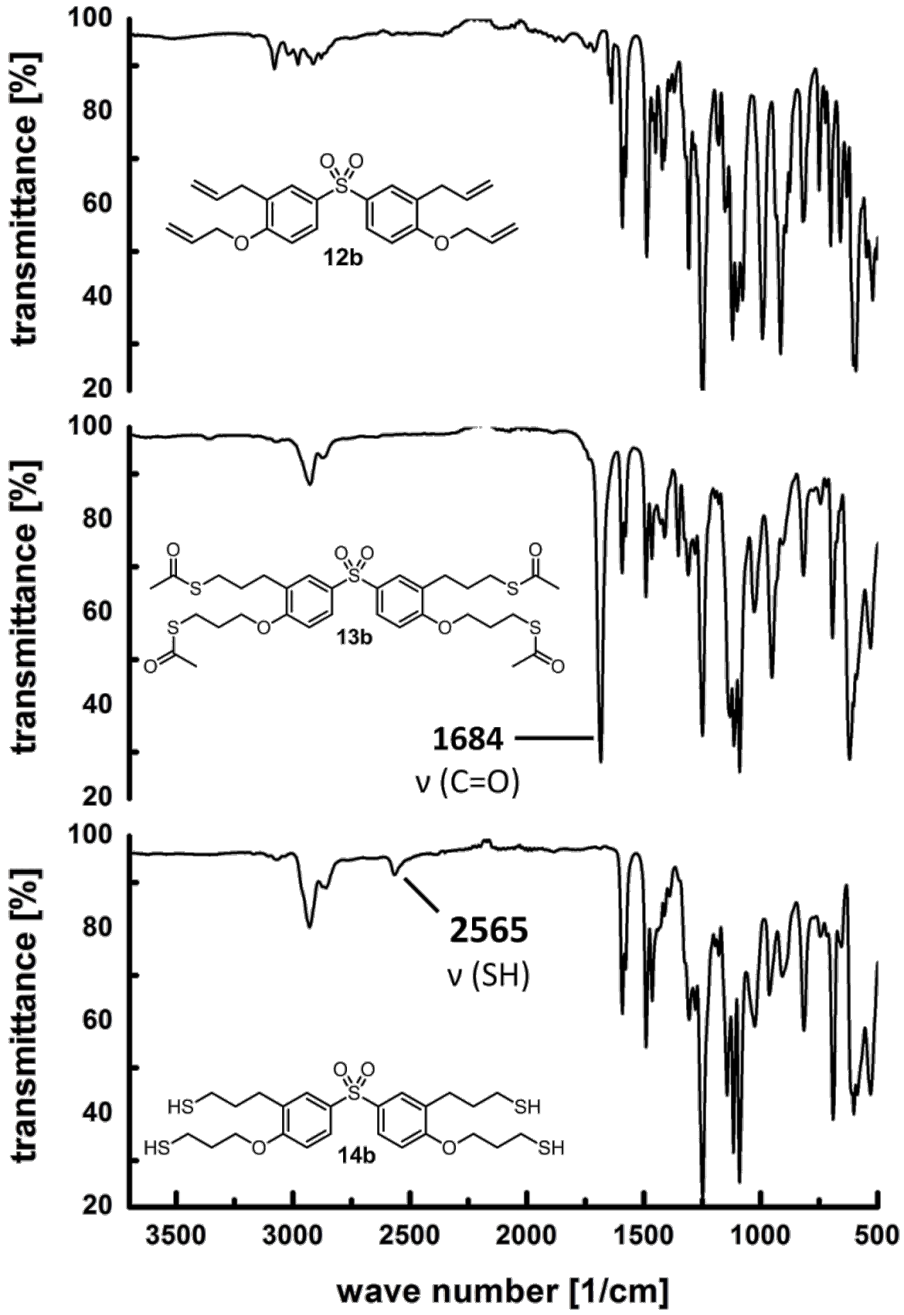
FTIR spectra of compounds **12b**, **13b** and **14b**.

**Figure 2:**
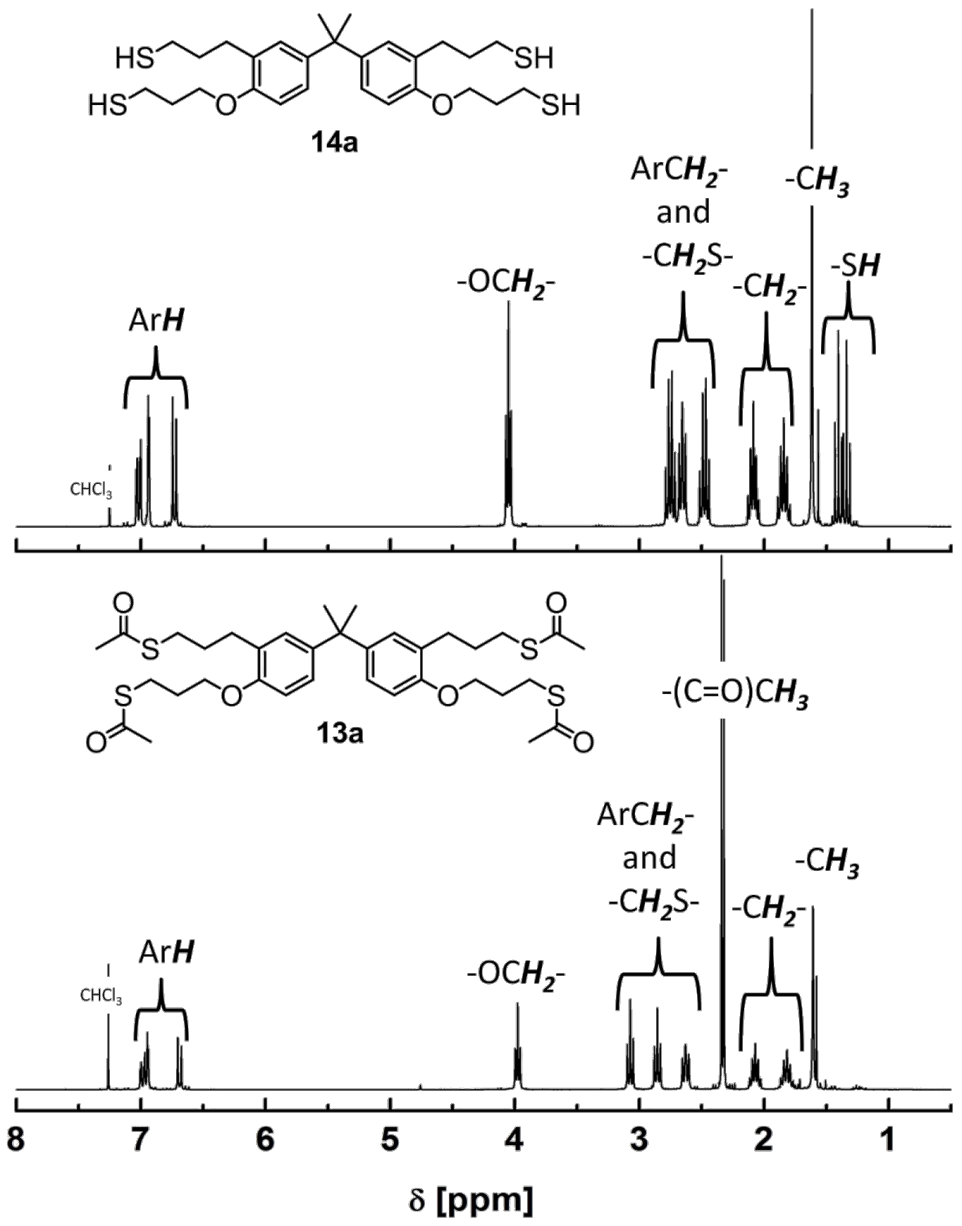
Top: ^1^H NMR spectrum of 3,3’-(propane-2,2-diylbis(2-(3-mercaptopropoxy)-5,1-phenylene))bis(propane-1-thiol) (**14a**) in CDCl_3_ (300 MHz, rt); bottom: ^1^H NMR spectrum of *S*,*S*’-((propane-2,2-diylbis(2-(3-(acetylthio)propoxy)-5,1-phenylene))bis(propane-3,1-diyl)) diethanethioate (**13a**) in CDCl_3_ (300 MHz, rt).

The second route for the synthesis of the allyl-modified precursors (**12a–c, 15**) proceeded via Claisen rearrangement of the allylaryl ethers and subsequent etherification, which was conducted in analogy to a procedure described in literature [40], as demonstrated in Scheme 2. Bisphenol A, bisphenol S and the trisphenol derivative 1,1,1-tris(4-hydroxyphenyl)ethane were chosen as basic structures. Subsequently, the successful radical addition of thioacetic acid (**8**) to the allyl-modified precursors (**7a,b**, **12a–c**, **15**) by use of 2,2’-azobis(2-methylpropionitrile) (AIBN) as the radical source was easily proven by the occurrence of the typical C=O valence vibration of the thioester (**9a,b**, **13a–c**, **16**) in the corresponding FTIR spectra at 1684 cm^−1^ (Figure 1). Additionally, in the range of 6.2 to 4.9 ppm signals corresponding with the presence of a double bond were no longer observed in the ^1^H NMR spectra. Simultaneously, the appearance of a signal at 2.4 to 2.3 ppm was observed, which corresponds with the methyl group of the thioester (see Figure 2).

The thioester derivatives **9a,b**, **13a–c** and **16** were successfully hydrolyzed under acidic conditions to obtain the thiols **10a,b, 14a–c** and **17** as viscous oils. The corresponding FTIR spectra showed the disappearance of the strong C=O valence vibration at 1684 cm^−1^ and the simultaneous appearance of the weak S–H valence vibration at approximately 2560 cm^−1^. Exemplarily, Figure 1 shows a comparison of the FTIR spectra of compound **12b**, **13b**, and **14b**. In Figure 2, a typical ^1^H NMR spectrum of the thioester **13a** and the thiol **14a** is shown (further ^1^H and ^13^C NMR spectra of all thiols can be found in Supporting Information File 1). Besides FTIR, ^1^H and ^13^C NMR spectroscopy, elemental analysis and mass spectrometry was conducted for detailed characterization of all products **10a,b**, **14a–c** and **17**.

**Scheme 3:**
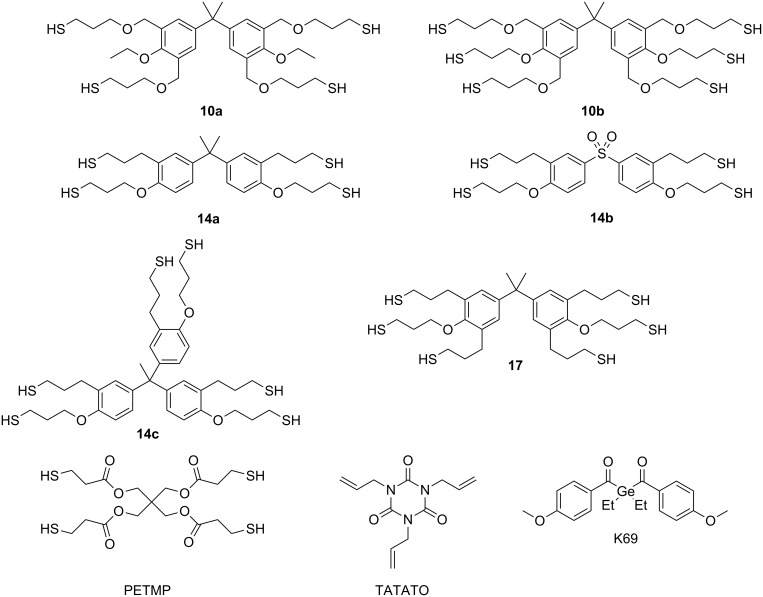
Chemical structures of components utilized in the present study.

**Mechanical properties of 10a,b, 14a–c and 17 employed in thiol–ene photopolymerizations.** In this study we measured the flexural strength, flexural modulus of elasticity and the conversion of unfilled thiol–ene samples containing thiol compounds **10a,b**, **14a–c** and **17** and TATATO (see Scheme 3) in stoichiometric amounts with respect to the functional groups. For evaluation of the mechanical performance of the phenol based thiols in photopolymerizations the widely used monomer PETMP was used as reference monomer [22–23,33]. As the initiating compound, the newly developed photoinitiator di(4-methoxy)benzoyl(diethyl)germane (K69) was utilized since recent studies showed an excellent performance of K69 in dental materials compared to campherchinone/4-(*N*,*N*-dimethylamino)benzoate initiation [41].

As evident from Table 1, all investigated thiol derivatives (**10b**, **14a–c** and **17**) – except for monomer **10a** – outperformed the reference monomer PETMP regarding flexural strength and flexural modulus of elasticity of the photopolymerized thiol–ene samples. For the derivatives with a propyl spacer, **14a–c** and **17**, the flexural strength varied between 121.8 MPa (**14a**) and 112.7 MPa (**17**) compared to 109.3 MPa for PETMP. For the samples containing **14a–c** and **17** no change in the flexural strength was observed after storing the samples in water for 24 hours at 37 °C. In contrast to that, the flexural strength of the PETMP containing sample decreased by 50% to a value of 47.8 MPa. Similar results were found for the modulus of elasticity: the PETMP sample showed a decrease from 3.2 GPa to 1.0 GPa after storage in water, whereas for **14a–c** and **17** values between 3.0 GPa and 2.8 GPa were found that slightly decreased to 2.8 GPa and 2.7 GPa after storage in water. Thus, as expected, the mechanical properties of the PETMP based sample were more effected by the storage in water, due to the more hydrophilic character of PETMP compared to **14a–c** and **17**.

**Table 1:** Flexural strength and flexural modulus of elasticity before and after storage in H_2_O as well as the conversion. All samples contained a stoichiometric amount of TATATO and the below-mentioned thiol, 0.5 wt % initiator (K69) and 0.04 wt % Q1301.

Thiol	Flexural strength [MPa]		Flexural modulus of elasiticity [GPa]		Conversion [%]	

	24 h rt	24 h H_2_O	24 h rt	24 h H_2_O	Thiol	Ene

PETMP	109.3 ± 2.6	47.8 ± 3.6	3.2 ± 0.2	1.0 ± 0.2	89.2 ± 4.2	83.9 ± 0.9
**10a**	29.1 ± 1.5	9.1 ± 0.8	0.4 ± 0.1	0.1 ± 0.02	97.2 ± 4.8	87.2 ± 3.0
**10b**	98.4 ± 8.5	87.9 ± 5.7	3.0 ± 0.2	2.6 ± 0.3	81.9 ± 1.7	83.9 ± 2.5
**14a**	121.8 ± 3.4	120.1 ± 2.8	3.0 ± 0.2	2.9 ± 0.2	66.4 ± 2.7	93.5 ± 2.0
**14b**	133.6 ± 6.4	132.2 ± 6.7	2.8 ± 0.2	2.9 ± 0.3	71.4 ± 2.0	61.9 ± 0.8
**14c**	127.8 ± 6.2	125 ± 12.0	3.0 ± 0.2	2.9 ± 0.2	68.2 ± 1.8	79.0 ± 1.0
**17**	112.7 ± 3.1	119.4 ± 3.6	2.9 ± 0.2	2.7 ± 0.1	76.2 ± 0.9	82.6 ± 0.8

Surprisingly, no significant effect of the degree of functionalization (four versus six thiol groups) on the mechanical properties of the cured sample was observed while comparing **14a** and **17**. Regarding **14a** and **14b**, no significant effect was found by changing the backbone from bisphenol A to bisphenol S. Monomer **10a**, incorporating four thiol-groups with a longer, more flexible and hydrophilic methoxypropyl spacer showed a significantly lower flexural strength of 29.1 MPa, and 9.1 MPa after water storage. For the flexural modulus values of 0.4 GPa and 0.1 GPa, respectively, were found. The introduction of two additional thiol groups in **10b** compared to **10a** improved the mechanical properties significantly.

**Figure 3:**
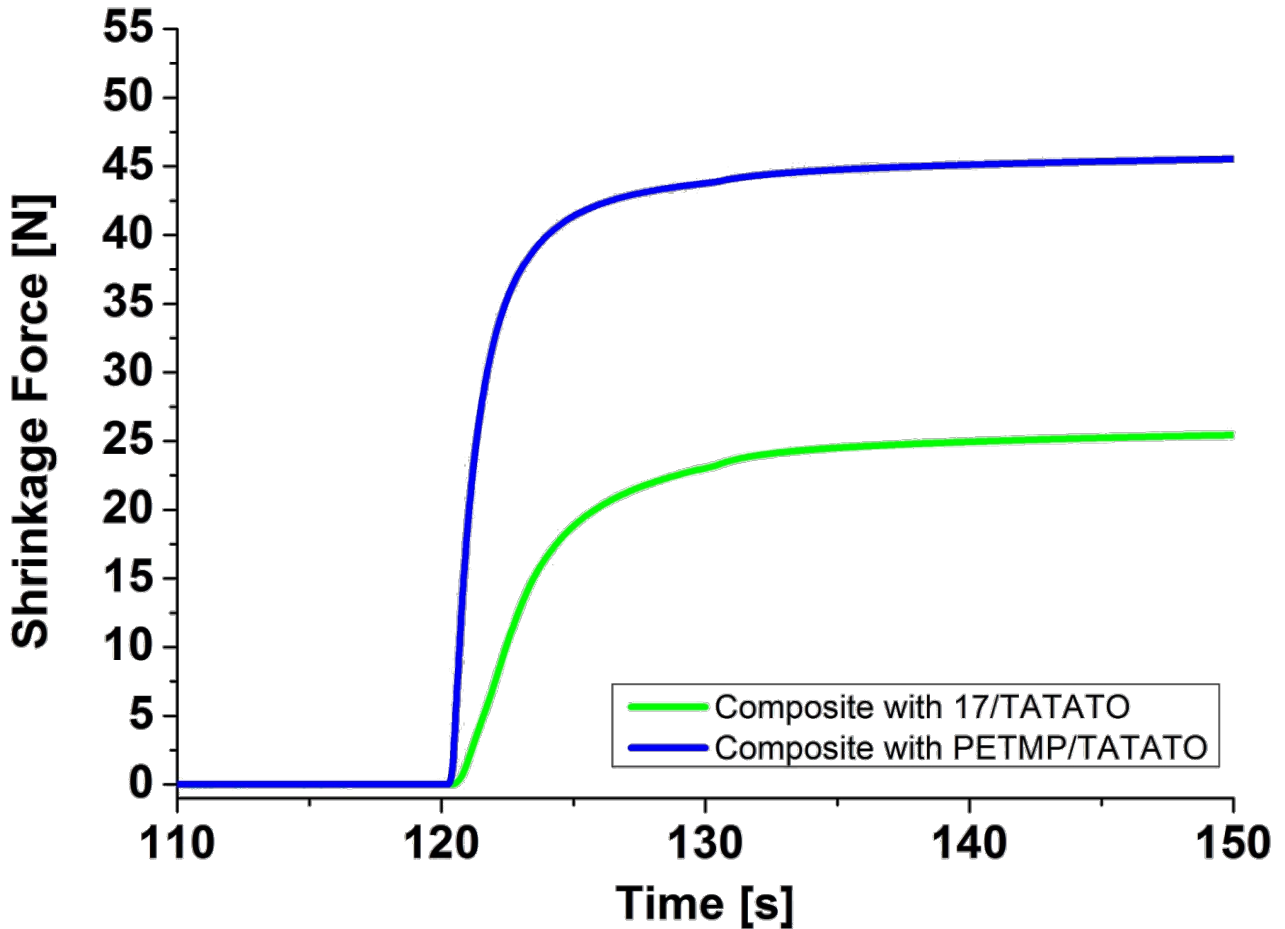
Development of the shrinkage force as function of time of dental composites containing 39.8 wt % organic matrix based stoichiometric amounts of TATATO and the thiol component (PETMP (blue line) or **17** (green line)), 59.98 wt % inorganic filler, 0.20 wt % initiator (K69) and 0.02 wt % Q1301. The exposure to light was initiated after 120 s.

Additionally, the performance of derivative **17** in a dental composite containing a stoichiometric amount with respect to the functional groups of TATATO was investigated and compared to a reference system containing PETMP/TATATO. The samples were prepared with a filler content of 59.98 wt %, an initiator content of 0.2 wt % and 0.02 wt % inhibitor. The experimental results are summarized in Table 2. For the PETMP/TATATO system values for the flexural strength of 86.9 MPa and 61.9 MPa after storage in water while for the flexural modulus of 4.9 GPa and 2.2 GPa after storage in water were found. For the **17/**TATATO system the flexural strength was found to be 101.5 MPa slightly decreasing to 97.0 MPa after storage in water. The corresponding flexural modulus was determined to 5.1 GPa and 4.2 GPa after storage in water. These results indicate that the mechanical properties of the composite containing **17** and TATATO as the organic matrix are higher than the reference system of PETMP/TATATO. Thus, after water storage the sample containing **17**/TATATO showed significantly improved values for the flexural strength as well as for the flexural modulus. Additionally, the shrinkage force was measured using a Zwick UPM. For the sample containing **17**/TATATO the shrinkage force (29.1 N) was found to be 40% lower compared to the reference system PETMP/TATATO (48.5 N), although the mechanical properties were even better (see Table 2 and Figure 3). The exothermic temperature was comparable for both systems. In contrast the exothermic time was longer for the system **17**/TATATO which indicates a slower polymerization kinetic (see Figure 3).

**Table 2:** Flexural strength and modulus of elasticity before and after storage in H_2_O, shrinkage force, exothermic setting time and exothermic setting temperature of dental composites containing 39.8 wt % organic matrix based on stoichiometric amounts of TATATO and the thiol component (PETMP respectively **17**), 59.98 wt % inorganic filler, 0.20 wt % initiator (K69) and 0.02 wt % Q1301.

Thiol	Flexural strength 24 h rt; (24 h H_2_O) [MPa]	Flexural modulus of elasticity 24 h rt; (24 h H_2_O) [GPa]	Shrinkage force [N]	Exothermic setting temperature (*T*_s_)^a^ [°C]	Exothermic setting time (*t*_s_)^b^ [s]

PETMP	86.9 ± 7.6; (61.9 ± 5.7)	4.9 ± 0.2; (2.2 ± 0.2)	48.5 ± 5.1	17 ± 2.1	5.0 ± 0.01
**17**	101.5 ± 5.8; (97.0 ± 6.4)	5.1 ± 0.1; (4.2 ± 0.2)	29.1 ± 1.2	13.7 ± 0.4	14.0 ± 1.0

^a^Exothermic setting temperature is defined as the highest temperature reached during the curing process; ^b^exothermic setting time is defined as the time needed to reach the highest temperature.

## Conclusion

In the present study we successfully synthesized and evaluated thiol-modified bis- and trisphenol derivatives for photopolymerizations with TATATO serving as the ene monomer. In comparison to the widely used ester derivatives based on 3-mercaptopropionic acid the presented thiol derivatives were ester-free and thus, hydrolytically stable, so that they overcome one of the main drawbacks of established thiols. Four of the six synthesized thiol derivatives outperformed the standard thiol PETMP regarding the flexural strength and flexural modulus of elasticity. To be utilizable for applications in dentistry, the mechanical performance after storage in water is of great importance. With respect to this aspect, the investigated thiols lead to an improvement of the flexural strength of up to 250% and up to 280% for the flexural modulus of elasticity compared to the initial values of the reference system PETMP/TATATO. Exemplarily, the performance of monomer **17** in a filled composite formulation was investigated and compared to a composite containing PETMP, still showing the excellent performance of the bisphenol monomer in matters of flexural strength, modulus of elasticity and shrinkage force.

## Supporting Information

General descriptions of materials and methods, the syntheses of the obtained compounds (**5**, **7a,b**, **9a,b**, **10a,b**, **11a–c**, **12a–c**, **13a–c**, **14a–c**, **15**, **16**, **17**) as well as analytical data (^1^H and ^13^C NMR, FTIR and EIMS analyses) of these compounds and copies of ^1^H and ^13^C NMR spectra of compounds (**10a,b**, **14a–c**, **17**) plus the ^1^H NMR spectra of compounds (**5**, **7a**, **7b**, **9a**, **9b**, **12c**, **13a–c**, **16**) are given.

File 1 Experimental part.
