# Supplementary material for: Investigations of thiol-modified phenol derivatives for the use in thiol–ene photopolymerizations
Source: Beilstein J Org Chem. 2014 Jul 29;10:1733–40. doi: 10.3762/bjoc.10.180 (PMC4142978; doi:10.3762/bjoc.10.180)
Supplement: File 1 — Experimental part. [file Beilstein_J_Org_Chem-10-1733-s001.pdf]

Supporting Information

for

Investigations of thiol-modified phenol derivatives for the use in thiol–ene photopolymerizations

Sebastian Reinelt[1], Monir Tabatabai[1], Urs Karl Fischer[2], Norbert Moszner[2], Andreas Utterodt[3] and Helmut Ritter[1]*

Address: [1]Heinrich-Heine-University Düsseldorf, Institute of Organic Chemistry and Macromolecular Chemistry, Department of Preparative Polymer Chemistry, Universitätsstraße 1, 40225 Düsseldorf, Germany, [2]Ivoclar Vivadent AG, Bendererstrasse 2, 9494 Schaan, Principality of Liechtenstein and [3]Heraeus Kulzer GmbH, Philipp-Reis-Straße 8, 61273 Wehrheim, Germany

*Corresponding author

Email: Helmut Ritter - h.ritter@uni-duesseldorf.de

Experimental part

A. Methods

^1H NMR and ^{13}C NMR measurements were performed using a Bruker Avance 300 or 600 spectrometer operating at 300 and 600 MHz for ^1H- and at 75 and 150 MHz for ^{13}C-experiments at room temperature. The signals of the uncomplete deuterated solvents or tetramethylsilane were used as internal standard. For elemental analyses the elemental analyzer vario MICRO Cube (Elementar analysensyteme GmbH, Germany) was used. Fourier transformation infrared spectroscopy (FT-IR) was performed on a Nicolet 6700 FT-IR spectrometer equipped with a diamond single bounce ATR accessory. The measurements were performed in the range of 4000–300 cm^{-1} at room temperature. GC/MS (EI) spectra were recorded on a GC/MS-System comprising a triple-quadrupol mass spectrometer Finnigan Trace DSQ and a Finnigan Trace GC Ultra. MS (EI) spectra were recorded on triple-quadrupol mass spectrometer Finnigan TSQ 7000 and MS (ESI) spectra on an Ion-Trap-API Finningan LCQ Deca (Thermo Quest) mass spectrometer. Ionization was carried out by electrospray ionization. Matrix-assisted laser desorption-ionization time-of-flight mass spectrometry (MALDI-TOF-MS) was performed on a Bruker Ultraflex time of flight mass spectrometer. The measurements were carried out in the linear modus using a suitable matrix with a 337 nm N_2 laser. Thermal properties were determined using a Büchi Meltingpoint B-545 at a heating rate of 1 °C/min.

B. Materials

1,1,1-Tris(4-hydroxyphenyl)ethane (Sigma-Aldrich, 99%), 1,3,5-triallyl-1,3,5-triazine-2,4,6-trione (Fluka, 97%), 2,2'-azobis(2-methylpropionitrile) (Acros Organics, 98%), 4,4'-(propane-2,2-diyl)diphenol (Alfa Aesar, 97%), 4,4`-sulfonyldiphenol (Acros Organics, 99%), allyl bromide (Acros Organics, 99%), bis(4-methoxybenzoyl)diethyl-germanium Ivocerin® (Ivoclar Vivadent AG, Liechtenstein), thioacetic acid (Acros Organics, 98%), formaldehyde solution (Grüssing, 37 wt% in H_2O), hydrochloric acid 37% (VWR, normapur), iodoethane

(Sigma Aldrich, 99%), N-nitrosophenylhydroxylamine aluminium salt Q-1301 (WAKO Pure Chemicals Industries Ltd., Osaka, Japan), pentaerythritoltetra(3-mercaptopropionate) (Sigma Aldrich, 95%), sodium hydride (Sigma Aldrich, 60%), sodium hydroxide (AppliChem, p.a.), potassium carbonate (Sigma Aldrich, p.a.) were used as received. Chloroform-d (99.8 atom% D) and dimethylsulfoxide (DMSO)-d_6 (99.9 atom% D) were obtained from Deutero GmbH (Germany). As not stated different – all other chemicals were of analytical grade and were used as received without any further purification. All solvents were dried by standard methods. Column chromatography was performed using Fluka silica gel 60 (230-400 mesh) and thin layer chromatography (TLC) of the products using Merck silica 60 F_{254} plates. 2,2-Bis(3,5-bishydroxymethyl-4-hydroxyphenyl)propane (**3**) was synthesized according to established literature procedures[1].

Commercial products were used as fillers: fumed silica Ox-50 (size of primary particles: 40 nm, Degussa, Frankfurt, Germany) and YbF_3 (mean particle size: 800 nm, Sukgyung, Korea). Ox-50 was silanized by mixing it with 1.0 wt % of water and 5.0 wt % of 3-methylacryloxypropyltrimethoxysilane (MPTS, Union Carbide) at room temperature over a period of 2 h, and drying the modified filler at 50 °C for 4 days.

C. Synthesis of the precursors (5 and 7a,b)

2,2-Bis(3,5-bis(hydroxymethyl)-4-ethoxyphenyl)propane (5). 2,2-Bis(3,5-bis(hydroxyl-methyl)-4-hydroxyphenyl)propane **3** (19.90 g, 60 mmol), iodoethane **4** (30.41 g, 195 mmol) and potassium carbonate (16.58 g, 120 mmol) were dissolved in 300 mL acetone. The mixture was refluxed under a nitrogen atmosphere until the reaction was completed (as monitored by TLC). Then 100 mL of deionized water was added and extracted with ethylacetate (3 x 200

mL). The combined organic extracts were washed with 1N NaOH-solution (2 x 100 mL) and brine (2 x 100 mL), dried over anhydrous $MgSO_4$, filtered and concentrated under reduced pressure. Purification by column chromatography with ethylacetate (R_f=0.48) as eluent afforded the desired product **5** (14.50 g, 63%) as white solid. mp: 123 °C; ^{1}H NMR (300 MHz, DMSO-d$_6$, δ [*ppm*]): 7.22 (s, 4H, Ar***H***), 5.06 (t, $^3J_{HH}$=5.5 Hz, 4H, CH_2O***H***), 4.49 (d, $^3J_{HH}$=5.5 Hz, 8H, C***H₂***OH), 3.80 (q, $^3J_{HH}$=7.0 Hz, 4H, OC***H₂***), 1.61 (s, 6H, C(C***H₃***)₂), 1.29 (t, $^3J_{HH}$=7.0 Hz, 6H, C***H₃***); ^{13}C NMR (75 MHz, DMSO-d$_6$, δ [*ppm*]): 151.23 (Ar***C***), 145.38 (Ar***C***), 134.06 (Ar***C***), 124.92 (Ar***C***), 69.44 (O***C***H_2)**,** 59.09 (***C***H_2OH), 42.04 (***C***$(CH_3)_2$), 31.10 (C(***C***$H_3)_2$), 15.74 (CH_2***C***H_3); FT-IR: *ν* = 3233 (m, ν_{OH}), 2972 (m, ν_{CH3}), 2930 (m, ν_{CH2}), 2870 (m, ν_{CH3}), 1591 (w), 1463 (s, δ_{CH3}), 1388 (m, δ_{CH3}), 1361 (m), 1222 (m), 1183 (vs), 1106 (m), 1065(s), 1030 (vs) cm^{-1}; MS (EI) *m*/*z* (%): 404 (24) [M$^+$], 390 (24), 389 (100), 295 (11), 43 (32); Anal. calc. for $C_{23}H_{32}O_6$: C 68.29, H 7.97, found: C 67.07, H 7.65.

5,5'-(Propane-2,2-diyl)bis(1,3-bis(allyloxy)methyl)-2-ethoxybenzene (7a). 2,2-Bis(3,5-bis(hydroxymethyl)-4-ethoxyphenyl)propane **5** (11.33 g, 28 mmol) was dissolved in dry DMF (150 mL) under a nitrogen atmosphere. The solution was cooled down with an ice bath to 0 °C and sodium hydride (6.72 g, 168 mmol, 60% oil dispersion) was added slowly to the solution. After stirring the suspension for 60 min allyl bromide **6** (20.33 g, 168 mmol) was added dropwise. The solution was stirred for 48 h at room temperature and the solvent was removed under reduced pressure. To the residual oil 200 mL of deionized water were added and extracted with dichloromethane (3 x 150 mL). The combined organic extracts were washed with 1N HCl-solution (2 x 70 mL) and brine (2 x 70 mL), dried over anhydrous $MgSO_4$, filtered and concentrated under reduced pressure. Purification by column chromatography with ethylacetate/petrolether 1/8 (R_f=0.35) as eluent afforded the desired product **7a** (14.4 g, 91%) as clear oil. ^{1}H NMR (300 MHz, $CDCl_3$, δ [*ppm*]): 7.20 (s, 4H, Ar***H***), 5.88 (ddt, $^3J_{HH}$=5.6 Hz / $^3J_{HH}$=10.4 Hz / $^3J_{HH}$=17.2 Hz, 4H, C***H***=CH_2), 5.22 (dq,

$^3J_{HH}$=17.2 Hz, 4H, CH=C$\boldsymbol{H_2}$), 5.13 (dq, $^3J_{HH}$=10.4 Hz, 4H, CH=C$\boldsymbol{H_2}$), 4.49 (s, 8H, ArC$\boldsymbol{H_2}$), 3.96 (dt, $^4J_{HH}$=1.4 Hz / $^3J_{HH}$=5.6 Hz, 8H, OC$\boldsymbol{H_2}$), 3.88 (q, $^3J_{HH}$=7.0 Hz, 4H, OC$\boldsymbol{H_2}$), 1.65 (s, 6H, C(C$\boldsymbol{H_3}$)$_2$), 1.36 (t, $^3J_{HH}$=7.0 Hz, 6H, CH$_2$C$\boldsymbol{H_3}$); ^{13}C NMR (75 MHz, CDCl$_3$, δ [*ppm*]): 154.03 (Ar***C***), 146.14 (Ar***C***), 134.88 (***C***H=CH$_2$), 130.65 (Ar***C***), 128.22 (Ar***C***), 117.13 (CH=***C***H$_2$), 71.24 (O***C***H$_2$), 70.91 (ArO***C***H$_2$), 67.44 (***C***H$_2$O), 42.40 (***C***(CH$_3$)$_2$), 31.00 (C(***C***H$_3$)$_2$), 15.77 (CH$_2$***C***H$_3$); FT-IR: ν = 3079 (w, ν_{CH}), 3012 (w, ν_{ArH}), 2973 (m, ν_{CH3}), 2927 (s, ν_{CH2}), 2855 (s, ν_{CH2}), 1647 (m, $\nu_{C=C}$), 1468 (m, δ_{CH2}), 1420 (w, δ_{CH2}), 1386 (m, δ_{CH3}), 1354 (m, δ_{CH3}), 1257 (m), 1225 (m), 1185 (s), 1080 (vs), 1032 (vs), 989 (s, $\delta_{-CH=H2}$), 919 (vs, $\delta_{-CH=H2}$) cm^{-1}; MALDI-TOF-MS: *m/z$_{found}$:* 564.3 (M+Na$^+$), 587.3 (M+K$^+$), *m/z$_{calc}$:* 564.35 (M+Na$^+$); Anal. calc. for $C_{35}H_{48}O_6$: C 74.44, H 8.57, found: C 73.83, H 8.69.

5,5'-(Propane-2,2-diyl)bis(2-(allyloxy)-1,3-bis((allyloxy)methyl)benzene (**7b**). The product **7b** was obtained by the same procedure as stated for the synthesis of **7a** starting from 2,2-bis(3,5-bis(hydroxymethyl)-4-hydroxyphenyl)propane **3** (13.07 g, 37.5 mmol), sodium hydride (13.49 g, 337.5 mmol, 60% oil dispersion) and allyl bromide **6** (40.83 g, 337.5 mmol). Purification by column chromatography with ethylacetate/n-hexane 1/9 (R_f=0.47) as eluent afforded the desired product **7b** (10.75 g, 49%) as clear oil. ^{1}H NMR (300 MHz, CDCl$_3$, δ [*ppm*]): 7.22 (s, 4H, Ar***H***), 6.08 (ddt, $^3J_{HH}$=5.4 Hz / $^3J_{HH}$=10.6 Hz / $^3J_{HH}$=17.2 Hz, 2H, C***H***=CH$_2$), 5.90 (ddt, $^3J_{HH}$=5.6 Hz / $^3J_{HH}$=10.4 Hz / $^3J_{HH}$=17.2 Hz, 4H, C***H***=CH$_2$), 5.46 – 5.12 (m, 12H, CH=C$\boldsymbol{H_2}$), 4.50 (s, 8H, ArC$\boldsymbol{H_2}$), 4.41 (dt, $^4J_{HH}$=1.4 Hz / $^3J_{HH}$=5.4 Hz, 4H, OC$\boldsymbol{H_2}$), 3.99 (dt, $^4J_{HH}$=1.4 Hz / $^3J_{HH}$=5.6 Hz, 8H, OC$\boldsymbol{H_2}$), 1.67 (s, 6H, C(C$\boldsymbol{H_3}$)$_2$); ^{13}C NMR (75 MHz, CDCl$_3$, δ [*ppm*]): δ = 153.96 (Ar***C***), 146.37 (Ar***C***), 134.88 (***C***H=CH$_2$), 134.21 (***C***H=CH$_2$), 130.79 (Ar***C***), 128.42 (Ar***C***), 117.13 (CH=***C***H$_2$), 116.94 (CH=***C***H$_2$), 76.02 (O***C***H$_2$), 71.33 (O***C***H$_2$), 67.56 (Ar***C***H$_2$) , 42.45 (***C***(CH$_3$)$_2$), 31.05 (C(***C***H$_3$)$_2$); FT-IR: ν = 3080 (w, ν_{CH}), 3006 (w, ν_{ArH}), 2968 (w, ν_{CH3}), 2935 (w, ν_{CH2}), 2858 (m, ν_{CH2}), 1647 (w, $\nu_{C=C}$), 1477 (m, δ_{CH2}), 1422 (m, δ_{CH2}), 1386 (w), 1354 (m, δ_{CH3}), 1290 (m), 1258 (w), 1183 (s), 1078 (vs,

ν), 986 (vs, $\delta_{-CH=H2}$), 919 (vs, $\delta_{-CH=H2}$) cm^{-1}; MALDI-TOF-MS: m/z_{found}: 611.3 ($M+Na^+$), 627.3 ($M+K^+$), m/z_{calc}: 611.33 ($M+Na^+$); Anal. calc. for $C_{37}H_{48}O_6$: C 75.48, H 8.22, found: C 73.81, H 7.75.

D. General procedure for the allylation and Claisen rearrangement of bisphenol derivatives

In a three-neck round-bottom flask the bisphenol derivative (**1a-c**) and potassium carbonate (0.9–1.5 equivalents per hydroxy group) were dissolved in acetone (approx. 10 wt% regarding the bisphenol component). After heating the white suspension to reflux for 60 minutes under a nitrogen atmosphere, allyl bromide **6** (1.3–1.5 equivalents per hydroxy group) was added through a dropping funnel. The mixture was refluxed until the reaction was completed (as monitored by TLC). Then deionized water was added and extracted three times with diethylether. The combined organic extracts were washed three times with 1N NaOH-solution and brine, dried over anhydrous $MgSO_4$, filtered and concentrated under reduced pressure.

4,4'-(Propane-2,2-diyl)bis((allyloxy)benzene) (**11a**). The product **11a** was synthesized by the general procedure as stated above. The product **11a** (36.5 g, 99% [Lit: 98%][2]) was obtained as clear liquid and used without further purification. ^1H NMR (300 MHz, $CDCl_3$, δ [*ppm*]): 7.12 (d, $^3J_{HH}$=8.9 Hz, 4H, Ar***H***), 6.80 (d, $^3J_{HH}$=8.9 Hz, 4H, Ar***H***), 6.03 (ddt, $^3J_{HH}$=5.3 Hz / $^3J_{HH}$=10.6 Hz / $^3J_{HH}$= 17.2 Hz, 2H, C***H***=CH_2), 5.39 (dq, $^3J_{HH}$=17.2 Hz, 2H, CH=C***H₂***), 5.25 (dq, $^3J_{HH}$=10.6 Hz, 2H, CH=C***H₂***), 4.48 (dt, $^3J_{HH}$=5.6 Hz / $^4J_{HH}$=1.5 Hz, 4H, OC***H₂***), 1.62 (s, 6H, C(C***H₃***)₂); ^{13}C NMR (75 MHz, $CDCl_3$, δ [*ppm*]): 156.60 (Ar***C***), 143.42 (Ar***C***), 133.69 (***C***H=CH_2), 127.86 (Ar***C***), 117.66 (CH=***C***H_2), 114.21 (Ar***C***), 68.93 (O***C***H_2), 41.83 (***C***(CH_3)₂), 31,22 (C(***C***H_3)₂); GC/MS (EI) *m/z* (%): 308 (24) [M^+], 294 (25) [$M-CH_3^+$], 293 (100) [$M-CH_3^+$], 211 (12).

4,4'-Sulfonylbis((allyloxy)benzene) (**11b**). The product **11b** was obtained by the general procedure as stated above. The product **11b** (40.5 g, 94% [Lit: 91%][3]) was obtained as clear solid and used without further purification. mp: 143 – 144 °C [Lit: 143 – 146 °C][3,4]; ^{1}H NMR (300 MHz, DMSO-d$_6$, δ [*ppm*]): 7.84 (d, $^3J_{HH}$=9.0 Hz, 4H, Ar***H***), 7.11 (d, $^3J_{HH}$=9.0 Hz, 4H, Ar***H***), 6.01 (ddt, $^3J_{HH}$=5.2 Hz / $^3J_{HH}$= 10.5 Hz / $^3J_{HH}$= 17.3 Hz, 2H, C***H***=CH$_2$), 5.37 (dq, $^3J_{HH}$=17.3 Hz, 2H, CH=C***H$_2$***), 5.26 (dq, $^3J_{HH}$=10.5 Hz, 2H, CH=C***H$_2$***), 4.63 (dt, $^3J_{HH}$=5.2 Hz / $^4J_{HH}$=1.5 Hz, 4H, OC***H$_2$***); ^{13}C NMR (75 MHz, DMSO-d$_6$, δ [*ppm*]): 161.78 (Ar***C***), 133.55 (Ar***C***), 132,84 (***C***H=CH$_2$), 129,31 (Ar***C***), 118.08 (CH=***C***H$_2$), 115.48 (Ar***C***), 68.66 (O***C***H$_2$); MS (EI) *m*/*z* (%): 332 (6) [M$^+$], 331 (18) [M$^+$], 330 (86) [M$^+$], 197 (23), 181 (13), 133 (51), 131 (14), 105 (15), 41 (100), 39 (12).

4,4',4''-(Ethane-1,1,1-triyl)tris((allyloxy)benzene) (**11c**). The product **11c** was obtained by the general procedure as stated above. The product **11c** (31.5 g, 98% [Lit: 70%][5]) was isolated as pale yellow liquid and used without further purification. ^{1}H NMR (300 MHz, CDCl$_3$, δ [*ppm*]): 7.00 (d, $^3J_{HH}$=8.9 Hz, 6H, Ar***H***), 6.82 (d, $^3J_{HH}$=8.9 Hz, 6H, Ar***H***), 6.07 (ddt, $^3J_{HH}$=5.3 Hz / $^3J_{HH}$=10.6 Hz / $^3J_{HH}$=17.2 Hz, 3H, C***H***=CH$_2$), 5.42 (dq, $^3J_{HH}$=17.2 Hz, 3H, CH=C***H$_2$***), 5.28 (dq, $^3J_{HH}$=10.6 Hz, 3H, CH=C***H$_2$***), 4.52 (dt, $^3J_{HH}$=5.3 Hz / $^4J_{HH}$=1.5 Hz, 6H, OC***H$_2$***), 2.12 (s, 3H, C(C***H$_3$***)); ^{13}C NMR (75 MHz, CDCl$_3$, δ [*ppm*]): 156.79 (Ar***C***), 142.11 (Ar***C***), 133.62 (***C***H=CH$_2$), 129.76 (Ar***C***), 117.75 (CH=***C***H$_2$), 114.01 (Ar***C***), 68.93 (O***C***H$_2$), 50.77 (***C***(CH$_3$)), 30.93 (C(***C***H$_3$)); MS (EI) *m*/*z* (%): 427 (3) [M$^+$], 426 (9) [M$^+$], 412 (30), 411 (100), 293 (10), 41 (10).

4,4'-(Propane-2,2-diyl)bis(2-allyl-1-(allyloxy)benzene) (**12a**). 4,4'-(Propane-2,2-diyl)bis-((allyloxy)benzene) **11a** (33.93 g, 110 mmol), was heated under a nitrogen atmosphere at 220 °C for 4 hours. The product was cooled down to room temperature. The subsequent allylation was afforded by the general procedure stated above. The product **12a** (41.5 g, 97% [Lit: 85%][4]) was obtained as clear liquid and used without further purification. ^{1}H NMR

(300 MHz, $CDCl_3$, δ [*ppm*]): 7.04 – 6.96 (m, 4H, Ar***H***), 6.71 (d, $^3J_{HH}$=8.3 Hz, 4H, Ar***H***), 6.10 – 5.89 (m, 4H, C***H***=CH_2), 5.40 (dq, $^3J_{HH}$=17.3 Hz, 2H, CH=C$\boldsymbol{H_2}$), 5.22 (dq, $^3J_{HH}$=10.6 Hz, 2H, CH=C$\boldsymbol{H_2}$), 5.05 – 4.94 (m, 4H, CH=C$\boldsymbol{H_2}$), 4.49 (dt, $^3J_{HH}$=5.0 Hz / $^4J_{HH}$=1.6 Hz, 4H, OC$\boldsymbol{H_2}$), 3.37 (d, $^3J_{HH}$=6.6 Hz, 4H, ArC$\boldsymbol{H_2}$), 1.62 (s, 6H, C(C$\boldsymbol{H_3}$)$_2$); ^{13}C NMR (75 MHz, $CDCl_3$, δ [*ppm*]): 154.29 (Ar***C***), 143.27 (Ar***C***), 137.40 (***C***H=CH_2), 133.92 (***C***H=CH_2), 128.67 (Ar***C***), 128.20 (Ar-***C***), 125.57 (Ar***C***), 116.88 (CH=***C***H_2), 115.29 (CH=***C***H_2), 111.18 (Ar***C***), 68.96 (O***C***H_2), 41.90 (***C***(CH_3)$_2$), 34.88 (Ar***C***H_2), 31.34 (C(***C***H_3)$_2$); FT-IR: *ν* = 3078 (w, ν_{CH}), 3009 (w, ν_{ArH}), 2967 (m, ν_{CH3}), 2929 (w, ν_{CH2}), 2908 (w), 2869 (w, ν_{CH2}), 1638 (m, $\nu_{C=C}$), 1606 (w, $\nu_{C=C}$), 1497 (vs, $\nu_{C=C}$), 1455 (m, δ_{CH2}), 1424 (m, δ_{CH2}), 1409 (m), 1382 (w, δ_{CH3}), 1361 (m, δ_{CH3}), 1299 (m), 1242 (vs), 1226 (vs), 1183 (m), 1128 (s), 1032 (s), 995 (s, $\delta_{-CH=H2}$), 911 (vs, $\delta_{-CH=H2}$), 808 (vs, δ_{-Ar-H}) cm^{-1}; MS (EI) *m/z* (%): 389 (10) [M^+], 388 (34) [M^+], 374 (29) [M^+-CH_3], 373 (100) [M^+-CH_3], 214 (13).

4,4'-Sulfonylbis(2-allyl-1-(allyloxy)benzene) (**12b)**. The product **12b** was synthesized by the same procedure as stated for **12a** starting from 4,4'-sulfonylbis((allyloxy)benzene) **11b** (39.65 g, 120 mmol), allyl bromide **6** (43.55 g, 360 mmol) and potassium carbonate (29.85 g, 216 mmol). The product **12b** (47.5 g, 97%) was obtained as white solid and used without further purification. mp: 81 °C; ^{1}H NMR (300 MHz, $CDCl_3$, δ [*ppm*]): 7.74 (dd, $^3J_{HH}$=8.6 Hz / $^4J_{HH}$=2.4 Hz, 2H, Ar***H***), 7.69 (d, $^4J_{HH}$=2.4 Hz, 2H, Ar***H***), 6.86 (d, $^3J_{HH}$=8.6 Hz, 2H, Ar***H***), 6.10 – 5.84 (m, 4H, C***H***=CH_2), 5.40 (dq, $^3J_{HH}$=17.3 Hz, 2H, CH=C$\boldsymbol{H_2}$), 5.29 (dq, $^3J_{HH}$=10.6 Hz, 2H, CH=C$\boldsymbol{H_2}$), 5.13 – 5.01 (m, 4H, CH=C$\boldsymbol{H_2}$), 4.57 (dt, $^3J_{HH}$=5.0 Hz / $^4J_{HH}$=1.6 Hz, 4H, OC$\boldsymbol{H_2}$), 3.40 (d, $^3J_{HH}$=6.7 Hz, 4H, ArC$\boldsymbol{H_2}$); ^{13}C NMR (75 MHz, $CDCl_3$, δ [*ppm*]): 159.75 (Ar***C***), 135.44 (***C***H=CH_2), 133.84 (***C***H=CH_2), 132.39 (Ar***C***), 130.22 (Ar***C***), 128.97 (Ar***C***), 127.60 (Ar***C***), 117.83 (CH=***C***H_2), 116.70 (CH=***C***H_2), 111.38 (Ar***C***), 69.05 (O***C***H_2), 34.31 (Ar***C***H_2); FT-IR: *ν* = 3078 (w, ν_{CH}), 3021 (w, ν_{ArH}), 2979 (m, ν_{CH2}), 2915 (m, ν_{CH2}), 2876 (w, ν_{CH2}), 1647 (w, $\nu_{C=C}$), 1638 (w, $\nu_{C=C}$), 1592 (m, $\nu_{C=C}$), 1579 (m, $\nu_{C=C}$), 1487 (s,

$\nu_{C=C}$), 1450 (m, δ_{CH2}), 1422 (m, δ_{CH2}), 1413 (m), 1370 (w), 1308 (s), 1249 (vs), 1180 (m), 1153 (m), 1120 (s), 1102 (s), 1078 (s), 992 (s, $\delta_{-CH=H2}$), 915 (vs, $\delta_{-CH=H2}$) cm^{-1}; MS (EI) *m*/*z* (%): 411 (29) [M^+], 410 (100) [M^+], 237 (51), 197 (10), 174 (13), 173 (59), 172 (11), 145 (18), 133 (15), 132 (35), 131 (55), 115 (13), 103 (23), 91 (11), 77 (11), 41 (53).

4,4',4''-(Ethane-1,1,1-triyl)tris(2-allyl-1-(allyloxy)benzene) (**12c**). The product **12c** was synthesized by the same procedure as stated for **12a** starting from 4,4',4''-(ethane-1,1,1-triyl)tris((allyloxy)benzene) **11c** (29.65 g, 69.50 mmol), allyl bromide **6** (33.64 g, 278 mmol) and potassium carbonate (25.93 g, 188 mmol). The product **12c** (37.5 g, 99%) was obtained as pale yellow oil and used without further purification. ^{1}H NMR (300 MHz, $CDCl_3$, δ [*ppm*]): 6.94 (d, $^4J_{HH}$=2.4 Hz, 3H, Ar***H***), 6.79 (dd, $^3J_{HH}$=8.6 Hz / $^4J_{HH}$=2.4 Hz, 3H, Ar***H***), 6.70 (d, $^3J_{HH}$=8.6 Hz, 3H, Ar***H***), 6.15 – 5.87 (m, 6H, C***H***=CH_2), 5.43 (dq, $^3J_{HH}$=17.3 Hz, 3H, CH=C$\boldsymbol{H_2}$), 5.26 (dq, $^3J_{HH}$=10.6 Hz, 3H, CH=C$\boldsymbol{H_2}$), 5.04 – 4.93 (m, 6H, CH=C$\boldsymbol{H_2}$), 4.52 (dt, $^3J_{HH}$=5.0 Hz / $^4J_{HH}$=1.6 Hz, 6H, OC$\boldsymbol{H_2}$), 3.35 (d, $^3J_{HH}$=6.5 Hz, 6H, ArC$\boldsymbol{H_2}$), 2.09 (s, 3H, C(C$\boldsymbol{H_3}$)); ^{13}C NMR (75 MHz, $CDCl_3$, δ [*ppm*]): 154.44 (Ar***C***), 142.06 (Ar***C***), 137.37 (***C***H=CH_2), 133.89 (***C***H=CH_2), 130.61 (Ar***C***), 128.04 (Ar***C***), 127.58 (Ar***C***), 116.94 (CH=***C***H_2), 115.25 (CH=***C***H_2), 110.85 (Ar***C***), 68.93 (O***C***H_2), 50.94 (***C***(CH_3)), 34.81 (Ar***C***H_2), 30.96 (C(***C***H_3)); FT-IR: *ν* = 3077 (w, ν_{CH}), 3009 (w, ν_{ArH}), 2977 (w, ν_{CH3}), 2929 (w, ν_{CH2}), 2909 (w), 2851 (w, ν_{CH2}), 1638 (w, $\nu_{C=C}$), 1605 (w, $\nu_{C=C}$), 1584 (w), 1496 (vs, $\nu_{C=C}$), 1455 (m, δ_{CH2}), 1423 (m, δ_{CH2}), 1409 (m), 1372 (w, δ_{CH3}), 1363 (w, δ_{CH3}), 1297 (m), 1245 (vs), 1223 (s), 1130 (s), 1022 (s), 995 (s, $\delta_{-CH=H2}$), 911 (vs, $\delta_{-CH=H2}$), 812 (s, δ_{-Ar-H}) cm^{-1}; MS (EI) *m*/*z* (%): 546 (8) [M^+], 532 (37) [M-CH_3^+], 531 (100) [M-CH_3^+], 373 (10), 41 (20); Anal. calc. for $C_{38}H_{42}O_3$: C 83.48, H 7.74 found: C 82.66, H 7.53.

5,5'-(Propane-2,2-diyl)bis(1,3-diallyl-2-(allyloxy)benzene) (**15**) [6]**.** The product **15** was synthesized by the same procedure as stated for **12a** starting from 4,4'-(propane-2,2-diyl)bis(2-allyl-1-(allyloxy)benzene) **12a** (18.46 g, 47.50 mmol), allyl bromide **6** (17.24 g,

142.50 mmol) and potassium carbonate (11.82 g, 85.50 mmol). Compound **15** was purified by column chromatography on silica gel with ethylacetate/n-hexane 1/19 (R_f=0.55) as eluent. The product **15** (15.1 g, 68%) was obtained as clear liquid. ^{1}H NMR (300 MHz, $CDCl_3$, δ [*ppm*]): 6.89 (s, 4H, Ar***H***), 6.14 – 5.86 (m, 6H, C***H***=CH_2), 5.40 (dq, $^3J_{HH}$=17.2 Hz, 2H, CH=C***H₂***), 5.22 (dq, $^3J_{HH}$=10.5 Hz, 2H, CH=C***H₂***), 5.03 – 4.93 (m, 8H, CH=C***H₂***), 4.28 (dt, $^3J_{HH}$=5.4 Hz / $^4J_{HH}$=1.5 Hz, 4H, OC***H₂***), 3.37 (m, 8H, ArC***H₂***), 1.61 (s, 6H, C(C***H₃***)₂); ^{13}C NMR (75 MHz, $CDCl_3$, δ [*ppm*]): 153.14 (Ar***C***), 146.37 (Ar***C***), 137.82 (***C***H=CH_2), 134.26 (***C***H=CH_2), 132.23 (Ar***C***), 127.27 (Ar***C***), 116.96 (CH=***C***H_2), 115.62 (CH=***C***H_2), 74.60 (O***C***H_2), 42.41 (***C***(CH_3)₂), 34.47 (Ar***C***H_2), 31.20 (C(***C***H_3)₂); FT-IR: ν = 3078 (w, ν_{CH}), 3000 (w, ν_{ArH}), 2975 (m, ν_{CH3}), 2929 (w, ν_{CH2}), 2909 (w, ν_{CH2}), 2863 (w, ν_{CH2}), 1638 (m, $\nu_{C=C}$), 1591 (w, $\nu_{C=C}$), 1499 (w), 1475 (s, δ_{CH2}), 1422 (m, δ_{CH2}), 1409 (m), 1383 (w, δ_{CH3}), 1361 (w, δ_{CH3}), 1275 (m), 1227 (m), 1172 (s), 1116 (m), 989 (vs, $\delta_{-CH=H2}$), 909 (vs, $\delta_{-CH=H2}$) cm^{-1}; MS (EI) *m*/*z* (%): 469 (35) [M^+], 468 (100) [M^+], 454 (10), 453 (30), 413 (13), 387 (10), 386 (13), 373 (11), 371 (20), 346 (12), 345 (37), 304 (10), 289 (12), 265 (18), 255 (28), 240 (15), 239 (74), 215 (13), 214 (11), 213 (21), 199 (25), 197 (11), 185 (10), 183 (10), 173 (12), 171 (14), 157 (14), 145 (14), 131 (13), 129 (14), 128 (16), 115 (10) 41 (18).

E. General procedure for the radical addition of thioacetic acid to the allylated bisphenol-derivatives

In a three-neck round-bottom flask the allylated bisphenol derivative (**7a,7b,12a-c,15**), thioacetic acid **8** (1.5–2.0 equivalents per double bond) and 2,2`-azobis(2-methylpropionnitrile) (AIBN) (0.05–0.06 equivalents per double bond) were dissolved in tetrahydrofuran (THF). The reaction mixture was purged with nitrogen for 30 minutes at ambient temperature and then stirred at 65 °C for 16 h under a nitrogen atmosphere. After cooling the reaction mixture in an ice bath to 0 °C, a 1N sodium carbonate solution was added and extracted three times with dichloromethane. The combined organic extracts were washed three times with a 1N sodium hydroxide solution and one time with brine, dried over $MgSO_4$, filtered and concentrated under reduced pressure. The residual smelling oil was purified via column chromatography on silica gel.

S,S’,S’’,S’’’-((Propane-2,2-diylbis(2-ethoxybenzene-5,3,1-triyl)tetrakis(methylene)-tetrakis-(oxy))tetrakis(propane-3,1-diyl)) tetraethanethioate (9a). The product **9a** was obtained by the general procedure as stated above starting from 5,5’-(propane-2,2-diyl)bis(1,3-bis(allyloxy)methyl)-2-ethoxybenzene **7a** (15.25 g, 27.00 mmol), thioacetic acid **8** (18.09 g, 237.60 mmol), AIBN (1.06 g, 6.48 mmol) and THF (130 mL). Purification by column chromatography with ethylacetate/n-hexane 1/4 (R_f=0.27) as eluent afforded the desired product **9a** (19.5 g, 83%) as pale yellow oil. ^{1}H NMR (300 MHz, $CDCl_3$, δ [*ppm*]): 7.13 (s, 4H, Ar***H***), 4.42 (s, 8H, ArC***H₂***), 3.82 (q, $^3J_{HH}$=7.0 Hz, 4H, OC$\boldsymbol{H_2}$$CH_3$), 3.42 (t, $^3J_{HH}$=6.1 Hz, 8H, OC$\boldsymbol{H_2}$), 2.85 (t, $^3J_{HH}$=7.2 Hz, 8H, C$\boldsymbol{H_2}$S), 2.25 (s, 12H, C$\boldsymbol{H_3}$), 1.83 – 1.70 (m, 8H, C$\boldsymbol{H_2}$), 1.62 (s, 6H, C(C$\boldsymbol{H_3}$)$_2$), 1.32 (t, $^3J_{HH}$= 7.0 Hz, 6H, $OCH_2C$$\boldsymbol{H_3}$); ^{13}C NMR (75 MHz, $CDCl_3$, δ [*ppm*]): 195.77 (S(***C***=O)CH_3), 153.75 (Ar***C***), 146.14 (Ar***C***), 130.63 (Ar***C***), 127.98 (Ar***C***), 70.88 (ArO***C***H_2), 68.80 (O***C***H_2), 68.14 (***C***H_2O), 42.40 (***C***$(CH_3)_2$), 31.05 (C(***C***H_3)$_2$), 30.66 (S(C=O)***C***H_3), 29.76 (***C***H_2S), 26.18 (***C***H_2), 15.81 (CH_2***C***H_3); FT-IR: ν =

2975 (w, ν_{CH3}), 2928 (w, ν_{CH2}), 2865 (m, ν_{CH2}), 1687 (vs, $\nu_{C=O}$), 1470 (m, δ_{CH2}), 1420 (m, δ_{CH2}), 1386 (m, δ_{CH3}), 1354 (m, δ_{CH3}), 1290 (w), 1264 (w), 1222 (w), 1184 (m), 1095 (vs), 1032 (s), 952 (s), 622 (vs) cm^{-1}; MALDI-TOF-MS: m/z_{found}: 891.4 ($M+Na^+$), 907.3 ($M+K^+$), m/z_{calcd}: 891.33 ($M+Na^+$); Anal. calc. for $C_{43}H_{64}O_{10}S_4$: C 59.42, H 7.42, S 14.76 found: C 59.15, H 7.14, S 14.82

S,S',S'',S'''-((((Propane-2,2-diylbis(2-(3-(acetylthio)propoxy)benzene-5,3,1-triyl))-tetrakis(methylene))tetrakis(oxy))tetrakis(propane-3,1-diyl)) tetraethanethioate (9b). The product **9b** was obtained by the general procedure as stated above starting from 5,5'-(propane-2,2-diyl)bis(2-(allyloxy)-1,3-bis(allyloxy)mehtyl)benzene **7b** (11.78 g, 20.00 mmol), thioacetic acid **8** (15.99 g, 210.00 mmol), AIBN (1.58 g, 4.81 mmol) and THF (100 mL). Purification by column chromatography with ethylacetate/n-hexane 1/2 (R_f=0.37) as eluent afforded the desired product **9b** (15.5 g, 73%) as pale yellow oil. ^1H NMR (300 MHz, $CDCl_3$, δ [*ppm*]): 7.13 (s, 4H, Ar***H***), 4.42 (s, 8H, ArC***H₂***O), 3.84 (t, $^3J_{HH}$=6.1 Hz, 4H, ArOC$\boldsymbol{H_2}$), 3.45 (t, $^3J_{HH}$=6.1 Hz, 8H, OC$\boldsymbol{H_2}$), 3.07 (t, $^3J_{HH}$=7.1 Hz, 4H, C$\boldsymbol{H_2}$S), 2.87 (t, $^3J_{HH}$=7.2 Hz, 8H, C$\boldsymbol{H_2}$S), 2.31 (s, 6H, S(C=O)C$\boldsymbol{H_3}$), 2.27 (s, 12H, S(C=O)C$\boldsymbol{H_3}$), 2.09 – 1.96 (m, 4H, C$\boldsymbol{H_2}$), 1.86 – 1.73 (m, 8H, C$\boldsymbol{H_2}$), 1.62 (s, 6H, C(C$\boldsymbol{H_3}$)$_2$); ^{13}C NMR (75 MHz, $CDCl_3$, δ [*ppm*]): 195.86 and 195.76 (S(***C***=O)CH_3), 153.56 (Ar***C***), 146.42 (Ar***C***), 130.69 (Ar***C***), 128.32 (Ar***C***), 73.45 (ArO***C***H_2), 68.96 (O***C***H_2), 68.27 (***C***H_2O), 42.50 (***C***(CH_3)$_2$), 31.12 (C(***C***H_3)$_2$), 30.82 (S(C=O)***C***H_3), 30.77 (S(C=O)***C***H_3), 30.43 (***C***H_2), 29.87 (***C***H_2), 26.26 (***C***H_2), 26.08 (***C***H_2); FT-IR: ν = 2961 (w, ν_{CH3}), 2929 (w, ν_{CH2}), 2866 (w, ν_{CH2}), 1685 (vs, $\nu_{C=O}$), 1594 (w), 1468 (m, δ_{CH2}), 1427 (m), 1386 (w), 1354 (m, δ_{CH3}) 1290 (w), 1264 (m), 1226 (m), 1182 (m), 1127 (s), 1096 (vs), 1022 (s), 952 (s), 622 (vs) cm^{-1}; MALDI-TOF-MS: m/z_{found}: 1067.4 ($M+Na^+$), 1083.4 ($M+K^+$), m/z_{calcd}: 1067.32 ($M+Na^+$); Anal. calc. for $C_{49}H_{72}O_{12}S_6$: C 56.29, H 6.94, S 18.40 found: C 55.27, H 6.62, S 19.06

S,S'*-((Propane-2,2-diylbis(2-(3-(acetylthio)propoxy)-5,1-phenylene))bis(propane-3,1-diyl)) diethanethioate (13a)**. The product **13a** was obtained by the general procedure as stated above starting from 4,4'-(propane-2,2-diyl)bis(2-allyl-1-(allyloxy)benzene) **12a** (29.14 g, 75.00 mmol), thioacetic acid **8** (34.25 g, 450.00 mmol), AIBN (2.46 g, 15.00 mmol) and THF (200 mL). Purification by column chromatography with ethylacetate/n-hexane 1/3 (R_f=0.53) as eluent afforded the desired product **13a** (35.8 g, 69%) as pale yellow oil. ^{1}H NMR (300 MHz, $CDCl_3$, δ [*ppm*]): 7.02 – 6.92 (m, 4H, ArH***), 6,69 (d, $^3J_{HH}$= 8.5 Hz, 2H, Ar***H***), 3.98 (t, $^3J_{HH}$=5.9 Hz, 4H, OC***H₂***), 3.07 (t, $^3J_{HH}$=7.1 Hz, 4H, C$\boldsymbol{H_2}$S), 2.85 (t, $^3J_{HH}$=7.2 Hz, 4H, C$\boldsymbol{H_2}$S), 2.63 (m, 4H, ArC$\boldsymbol{H_2}$), 2.34 and 2.32 (s, 12H, C$\boldsymbol{H_3}$), 2.14 – 2.01 (m, 4H, C$\boldsymbol{H_2}$), 1.90 -1.75 (m, 4H, C$\boldsymbol{H_2}$), 1.61 (s, 6H, C(C$\boldsymbol{H_3}$)$_2$); ^{13}C NMR (75 MHz, $CDCl_3$, δ [*ppm*]): 196.01 and 195.81 (S(***C***=O)CH_3), 154.50 (Ar***C***), 143.08 (Ar***C***), 128.98 (Ar***C***), 128.90 (Ar***C***), 125.39 (Ar***C***), 110.48 (Ar***C***), 66.12 (ArO***C***H_2), 41.75 (***C***(CH_3)$_2$), 31.26 (C(***C***H_3)$_2$), 30.80 (S(C=O)***C***H_3), 29.98 (***C***H_2), 29.83 (***C***H_2), 29.64 (***C***H_2), 29.05 (***C***H_2), 26.24 (***C***H_2); FT-IR: *ν* = 2962 (w, ν_{CH3}), 2926 (w, ν_{CH2}), 2867 (w, ν_{CH2}), 1685 (vs, $\nu_{C=O}$), 1607 (w, $\nu_{C=C}$), 1500 (s, $\nu_{C=C}$), 1470 (m, δ_{CH2}), 1415 (m), 1410 (m, δ_{CH2}), 1383 (w, δ_{CH3}), 1353 (m, δ_{CH3}), 1294 (w), 1243 (s), 1131 (s), 1105 (s), 1038 (m), 953 (s) cm^{-1}; MALDI-TOF-MS: m/z_{found}: 692.2 (M^+), 715.2 (M+Na^+), 731.2 (M+K^+), m/z_{calcd}: 715.22 (M+Na^+); Anal. calc. for $C_{35}H_{48}O_6S_4$: C 60.66, H 6.98 S 18.51 found: C 60.37, H 6.78, S 18.57

S,S'*-((Sulfonylbis(2-(3-(acetylthio)propoxy)-5,1-phenylene))bis(propane-3,1-diyl)) diethanethioate (13b).** The product **13b** was obtained by the general procedure as stated above starting from 4,4'-sulfonylbis(2-allyl-1-(allyloxy)benzene) **12b** (24.63 g, 60.00 mmol), thioacetic acid **8** (27.40 g, 360.00 mmol), AIBN (1.97 g, 12.00 mmol) and THF (200 mL). Purification by column chromatography with ethylacetate/n-hexane 1/1 (R_f=0.68) as eluent afforded the desired product **13b** (37.0 g, 86%) as pale yellow oil. ^{1}H NMR (300 MHz, $CDCl_3$, δ [*ppm*]): 7.74 (dd, $^3J_{HH}$=8.6 Hz / $^4J_{HH}$=2.4 Hz, 2H, ArH***), 7.66 (d, $^4J_{HH}$= 2.4 Hz, 2H,

Ar***H***), 6.85 (d, $^3J_{HH}$=8.6 Hz, 2H, Ar***H***), 4.04 (t, $^3J_{HH}$=6.0 Hz, 4H, OC***H₂***), 3.05 (t, $^3J_{HH}$=7.1 Hz, 4H, C***H₂***S), 2.87 (t, $^3J_{HH}$=7.2 Hz, 4H, C***H₂***S), 2.74 – 2.63 (m, 4H, ArC***H₂***), 2.34 (s, 12H, C***H₃***), 2.17 – 2.04 (m, 4H, C***H₂***), 1.90 - 1.79 (m, 4H, C***H₂***); ^{13}C NMR (75 MHz, $CDCl_3$, δ [*ppm*]): 195.79 and 195.60 (S(***C***=O)CH_3), 160.20 (Ar***C***), 133.85 (Ar***C***), 131.09 (Ar***C***), 129.09 (Ar***C***), 127.77 (Ar***C***), 111.07 (Ar***C***), 66.70 (O***C***H_2), 30.81 and 30.79 (S(***C***=O)CH_3), 29.59 (***C***H_2), 29.32 (***C***H_2), 28.91 (***C***H_2), 25.93 (***C***H_2); FT-IR: ν = 2929 (w, ν_{CH2}), 2870 (w, ν_{CH2}), 1684 (vs, $\nu_{C=O}$), 1593 (m, $\nu_{C=C}$), 1580 (m, $\nu_{C=C}$), 1491 (m, $\nu_{C=C}$), 1466 (m, δ_{CH2}), 1425 (w), 1411 (m, δ_{CH2}), 1353 (m), 1310 (m), 1281 (m), 1250 (vs), 1132 (s), 1114 (vs), 1090 (vs), 1028 (m), 952 (s), 816 (m) cm^{-1}; MALDI-TOF-MS: m/z_{found}: 715.1 ($M+H^+$), 737.1 ($M+Na^+$), m/z_{calcd}: 737.14 ($M+Na^+$); Anal. calc. for $C_{32}H_{42}O_8S_5$: C 53.75, H 5.92 S 22.42 found: C 53.78, H 5.77, S 22.65

S,S',S''*-((Ethane-1,1,1-triyltris(2-(3-(acetylthio)propoxy)benzene-5,1-diyl))tris(propane-3,1-diyl)) triethanethioate (13c)**. The product **13c** was obtained by the general procedure as stated above starting from 4,4',4''-(ethane-1,1,1-triyl)tris(2-allyl-1-(allyloxy)benzene) **12c** (19.41 g, 35.50 mmol), thioacetic acid **8** (24.32 g, 319.50 mmol), AIBN (1.75 g, 10.65 mmol) and THF (200 mL). Purification by column chromatography with ethylacetate/n-hexane 1/2 (R_f=0.54) as eluent afforded the desired product **13c** (27.0 g, 76%) as pale yellow oil. ^{1}H NMR (600 MHz, $CDCl_3$, δ [*ppm*]): 6.86 (d, $^4J_{HH}$=2.5 Hz, 3H, ArH***), 6.75 (d, $^3J_{HH}$=8.5 Hz / $^4J_{HH}$=2,5 Hz, 3H, Ar***H***), 6.66 (d, $^3J_{HH}$=8.5 Hz, 3H, Ar***H***), 3.98 (t, $^3J_{HH}$=5.9 Hz, 6H, OC***H₂***), 3.08 (t, $^3J_{HH}$=7.1 Hz, 6H, C***H₂***S), 2.83 (t, $^3J_{HH}$=7.2 Hz, 6H, C***H₂***S), 2.60 (m, 6H, ArC***H₂***), 2.34 and 2.31 (s, 18H, C***H₃***), 2.10 – 2.02 (m, 9H, C***H₂*** and C(C***H₃***)), 1,82 -1,76 (m, 6H, C***H₂***); ^{13}C NMR (75 MHz, $CDCl_3$, δ [*ppm*]): 195.99 and 195.84 (S(***C***=O)CH_3), 154.67 (Ar***C***), 141.80 (Ar***C***), 130.66 (Ar***C***), 128.81 (Ar***C***), 127.44 (Ar***C***), 110.23 (Ar***C***), 66.13 (O***C***H_2), 50.73 (***C***(CH_3)), 30.95 (C(***C***H_3)), 30.82 and 30.81 (S(C=O)***C***H_3), 29.96 (***C***H_2), 29.87 (***C***H_2), 29.65 (***C***H_2), 29.03 (***C***H_2), 26.26 (***C***H_2); FT-IR: ν = 2968 (w, ν_{CH3}), 2923 (w, ν_{CH2}), 2863 (w, ν_{CH2}),

1685 (vs, $\nu_{C=O}$), 1605 (w, $\nu_{C=C}$), 1498 (s, $\nu_{C=C}$), 1468 (m, δ_{CH2}), 1425 (m), 1410 (m, δ_{CH2}), 1352 (m, δ_{CH3}), 1293 (w), 1244 (s), 1131 (s), 1111 (vs), 1037 (m), 952 (s), 814 (m), 622 (vs) cm^{-1}; MALDI-TOF-MS: *m/z$_{found}$*: 987.3 ($M-CH_3^+$), 1025.3 ($M+Na^+$), *m/z$_{calcd}$*: 1025.29 ($M+Na^+$); Anal. calc. for $C_{50}H_{66}O_9S_6$: C 59.85, H 6.63 S 19.17 found: C 59.76, H 6.52, S 19.46

S,S'*,*S''*,*S'''*-(Propane-2,2-diylbis(2-(3-(acetylthio)propoxy)benzene-5,3,1-triyl))tetrakis-(propane-3,1-diyl)) tetraethanethioate (16)**. The product **16** was obtained by the general procedure as stated above starting from 5,5'-(propane-2,2-diyl)bis(1,3-diallyl-2-(allyloxy)benzene) **15** (27.56 g, 58.80 mmol), thioacetic acid **8** (40.28 g, 529.20 mmol), AIBN (2.90 g, 17.64 mmol) and THF (220 mL). Purification by column chromatography with ethylacetate/n-hexane 1/2 (R_f=0.68) as eluent afforded the desired product **16** (37.0 g, 86%) as pale yellow solid. mp: 66 °C; ^1H NMR (300 MHz, $CDCl_3$, δ [*ppm*]): 6.77 (s, 4H, ArH***), 3.75 (t, $^3J_{HH}$=6.0 Hz, 4H, OC***H$_2$***), 3.11 (t, $^3J_{HH}$=7.1 Hz, 4H, C***H$_2$***S), 2.83 (t, $^3J_{HH}$=7.2 Hz, 8H, C***H$_2$***S), 2.61 – 2.51 (m, 8H, ArC***H$_2$***), 2.33 and 2.30 (s, 18H, C***H$_3$***), 2.12 – 1.98 (m, 4H, C***H$_2$***), 1.85 – 1.71 (m, 8H, C***H$_2$***), 1.56 (s, 6H, C(C***H$_3$***)$_2$); ^{13}C NMR (75 MHz, $CDCl_3$, δ [*ppm*]): 195.91 and 195.87 (S(***C***=O)CH_3), 153.11 (Ar***C***), 146.49 (Ar***C***), 133.49 (Ar***C***), 128.80 (Ar***C***), 71.84 (ArO***C***H_2), 42.28 (***C***(CH_3)$_2$), 31.16 (C(***C***H_3)$_2$), 30.86 und 30.83 (S(C=O)***C***H_3), 30.72 (***C***H_2), 30.54 (***C***H_2), 29.66 (***C***H_2), 29.03 (***C***H_2), 26.21 (***C***H_2); FT-IR: *ν* = 3041 (w, ν_{ArH}), 2962 (w, ν_{CH3}), 2925 (m, ν_{CH2}), 2876 (w, ν_{CH2}), 2857 (w, ν_{CH2}), 1683 (vs, $\nu_{C=O}$), 1590 (w, $\nu_{C=C}$), 1467 (m, δ_{CH2}), 1451 (m), 1412 (m, δ_{CH2}), 1352 (m, δ_{CH3}), 1278 (m), 1262 (w), 1224 (m), 1177 (m), 1130 (s), 1103 (s), 1027 (m), 953 (s), 619 (vs) cm^{-1}; MALDI-TOF-MS: *m/z$_{found}$*: 925.3 ($M+H^+$), 947.3 ($M+Na^+$), *m/z$_{calcd}$*: 947.28 ($M+Na^+$); Anal. calc. for $C_{45}H_{64}O_8S_6$: C 58.41, H 6.97 S 20.79 found: C 58.14, H 6.89, S 20.64

F. General procedure for the hydrolysis of the thioesters (9a,b, 13a-c, 16)

In a three-neck round-bottom flask the thioester derivative (**9a,b, 13a-c, 16**) and aqueous concentrated hydrochloric acid (conc. hydrogen chloride (HCl)) (1.1 equivalents per thioester bond) were dissolved in a mixture of methanol and tetrahydrofuran (THF). The resulting solution was degassed by bubbling with nitrogen for 30 minutes and afterwards heated to 60 °C and kept at this temperature for 24 h under a nitrogen atmosphere. After cooling the reaction mixture deionized water was added and extracted three times with dichloromethane. The combined organic extracts were washed one time with a saturated sodium hydrogen carbonate solution and three times with brine. Depending on the purity the product was further purified via column chromatography on silica gel.

3,3',3'',3'''-(((Propane-2,2-diylbis(2-ethoxybenzene-5,3,1-triyl))tetrakis(methylene))-tetrakis(oxy)) tetrakis(propane-1-thiol) (10a). The product **10a** was obtained by the general procedure as stated above starting from **9a** (24.34 g, 28.00 mmol), conc. HCl (12.05 g), methanol (200 mL) and THF (20 mL). Purification by column chromatography with ethylacetate/n-hexane 1/4 (R_f=0.46) as eluent afforded the desired product **10a** (8.5 g, 44%) as pale yellow oil. ^{1}H NMR (300 MHz, $CDCl_3$, δ [*ppm*]): 7.14 (s, 4H, Ar***H***), 4.44 (s, 8H, ArC$\boldsymbol{H_2}$O), 3.85 (q, $^3J_{HH}$=7.0 Hz, 4H, OC$\boldsymbol{H_2}$$CH_3$), 3.45 (t, $^3J_{HH}$=6,1 Hz, 8H, OC$\boldsymbol{H_2}$), 2.88 (t, $^3J_{HH}$=7.2 Hz, 8H, C$\boldsymbol{H_2}$S), 1.85 - 1.75 (m, 8H, C$\boldsymbol{H_2}$), 1.64 (s, 6H, C(C$\boldsymbol{H_3}$)$_2$), 1.36 (t, $^3J_{HH}$= 7.0 Hz, 6H, CH_2C$\boldsymbol{H_3}$); ^{13}C NMR (75 MHz, $CDCl_3$, δ [*ppm*]): 153.86 (Ar***C***), 146.30 (Ar***C***), 130.82 (Ar***C***), 128.04 (Ar***C***), 71.00 (ArO***C***H_2), 68.42 (***C***H_2O), 68.26 (O***C***H_2), 42.53 (***C***$(CH_3)_2$), 34.09 (***C***H_2), 31.18 (C(***C***H_3)$_2$), 21.72 (***C***H_2SH), 15.96 (***C***H_3); FT-IR: ν = 2970 (m, ν_{CH3}), 2929 (m, ν_{CH2}), 2862 (m, ν_{CH2}), 2560 (w, ν_{SH}), 1594 (w, $\nu_{C=C}$), 1470 (m, δ_{CH2}) 1386 (m, δ_{CH3}), 1359 (m, δ_{CH3}), 1293 (m), 1267 (m), 1222 (m), 1183 (s), 1096 (vs), 1032 (vs), 889 (s) cm^{-1}; MS (EI) *m/z* (%): 685 (1) [M-CH_3^+], 516 (31), 443 (23), 427 (44), 383 (26), 353 (25), 352 (11), 351 (22), 339 (52), 337 (55), 335 (35), 333 (31), 325 (29), 323 (37), 309 (78), 307 (62), 189 (39), 161

(33), 159 (33), 119 (62), 75 (100), 41 (27); Anal. calc. for $C_{35}H_{56}O_6S_4$: C 59.96, H 8.05, S 18.29 found: C 60.18, H 7.82, S 17.97.

3,3',3'',3'''-((Propane-2,2-diylbis(2-(3-mercaptopropoxy)benzene-5,3,1-triyl)tetrakis-(methylene))tetrakis(oxy)) tetrakis(propane-1-thiol) (10b). The product **10b** was obtained by the general procedure as stated above starting from **9b** (15.16 g, 14.5 mmol), conc. HCl (9.36 g), methanol (150 mL) and THF (50 mL). Purification by column chromatography with ethylacetate/n-hexane 1/4 (R_f=0.41) as eluent afforded the desired product **10b** (5.1 g, 45%) as pale yellow oil. ^{1}H NMR (300 MHz, $CDCl_3$, δ [*ppm*]): 7.15 (s, 4H, Ar***H***), 4.45 (s, 8H, ArC***H₂***O), 3.92 (t, $^3J_{HH}$=6.0 Hz, 4H, ArOC***H₂***), 3.52 (t, $^3J_{HH}$=6.0 Hz, 8H, OC***H₂***), 2.77 (dt, $^3J_{HH}$=7.1 Hz and 8.0 Hz, 4H, C***H₂***SH), 2.55 (dt, $^3J_{HH}$=7.1 Hz and 8.0 Hz, 8H, C***H₂***SH), 2.15 – 2.00 (m, 4H, C***H₂***), 1.90 – 1.76 (m, 8H, C***H₂***), 1.64 (s, 6H, C(C***H₃***)₂), 1.47 (t, $^3J_{HH}$=8.0 Hz, 2H, S***H***), 1.30 (t, $^3J_{HH}$=8.0 Hz, 4H, S***H***); ^{13}C NMR (75 MHz, $CDCl_3$, δ [*ppm*]): 153.62 (Ar***C***), 146.49 (Ar***C***), 130.82 (Ar***C***), 128.35 (Ar***C***), 73.00 (ArO***C***H₂), 68.48 (O***C***H₂), 68.30 (***C***H₂O), 42.57 (***C***(CH₃)₂), 34.65 (***C***H₂), 34.08 (***C***H₂), 31.18 (C(***C***H₃)₂), 21.74 (***C***H₂SH), 21.55 (***C***H₂SH); FT-IR: *ν* = 2962 (w, ν_{CH3}), 2929 (m, ν_{CH2}), 2860 (m, ν_{CH2}), 2560 (w, ν_{SH}), 1591 (w), 1467 (m, δ_{CH2}), 1423 (w), 1383 (w, δ_{CH3}), 1360 (m, δ_{CH3}), 1286 (m), 1260 (m), 1224 (m), 1180 (s), 1093 (vs), 1020 (s) cm^{-1}; MS (ESI): m/z_{found}: 793.27 (M+H$^+$), $m/z_{calc.}$: 793.27 (M+H$^+$); Anal. calc. for $C_{37}H_{60}O_6S_6$: C 56.02, H 7.62, S 24.25 found: C 55.94, H 7.49, S 24.21.

3,3'-(Propane-2,2-diylbis(2-(3-mercaptopropoxy)-5,1-phenylene))bis(propane-1-thiol) (14a) [6]. The product **14a** was obtained by the general procedure as stated above starting from **13a** (35.69 g, 51.50 mmol), conc. HCl (22.17 g), methanol (200 mL) and THF (50 mL). Purification by column chromatography with dichloromethane/n-hexane (R_f=0.68) as eluent afforded the desired product **14a** (20.4 g, 76%) as pale yellow oil. ^{1}H NMR (300 MHz, $CDCl_3$, δ [*ppm*]): 7.02 (dd, $^3J_{HH}$=8.6 Hz / $^4J_{HH}$=2.5 Hz, 2H, Ar***H***), 6.94 (d, $^4J_{HH}$=2.5 Hz, 2H,

Ar***H***), 6.73 (d, $^3J_{HH}$=8.5 Hz, 2H, Ar***H***), 4.04 (t, $^3J_{HH}$=5.8 Hz, 4H, OC***H₂***), 2.79 – 2.70 (m, 4H, C***H₂***SH), 2.69 – 2.61 (m, 4H, ArC***H₂***), 2.53 – 2.42 (m, 4H, C***H₂***SH), 2.14 – 2.03 (m, 4H, C***H₂***), 1.90 – 1.77 (m, 4H, C***H₂***), 1.61 (s, 6H, C(C***H₃***)₂), 1.40 (t, $^3J_{HH}$=8.1 Hz, 2H, S***H***), 1.34 (t, $^3J_{HH}$=7.8 Hz, 2H, S***H***); ^{13}C NMR (75 MHz, $CDCl_3$, δ [*ppm*]): 154.54 (Ar***C***), 143.06 (Ar***C***), 129.08 (Ar***C***), 129.04 (Ar***C***), 125.22 (Ar***C***), 110.52 (Ar***C***), 65.56 (O***C***H₂), 41.75 (***C***(CH₃)₂), 34.31 (***C***H₂), 33.66 (***C***H₂), 31.26 (C(***C***H₃)₂), 29.43 (***C***H₂), 24.46 (***C***H₂), 21.63 (***C***H₂); FT-IR: *ν* = 3050 (w), 3025 (w, ν_{CH}), 2961 (m, ν_{CH3}), 2925 (m, ν_{CH2}), 2867 (w, ν_{CH2}), 2560 (w, ν_{SH}), 1606 (w, $\nu_{C=C}$), 1499 (vs, $\nu_{C=C}$), 1468 (m, δ_{CH2}), 1439 (m), 1383 (w, δ_{CH3}), 1360 (w, δ_{CH3}), 1294 (m), 1242 (vs), 1181 (m), 1154 (m), 1117 (m), 1034 (m), 810 (s) cm^{-1}; MS (EI) *m/z* (%): 525 (12) [M^+], 524 (36) [M^+], 511 (21), 510 (30), 509 (100), 450 (18), 437 (13), 436 (20), 435 (78), 362 (17), 361 (76), 327 (12), 287 (25), 213 (19), 209 (28), 207 (10), 193 (18), 179 (12), 175 (34), 159 (26), 147 (35), 135 (21), 133 (11), 119 (16), 107 (12), 75 (12), 47 (14), 41 (19); Anal. calc. for $C_{27}H_{40}O_2S_4$: C 61.79, H 7.68, S 24.44 found: C 61.99, H 7.62, S 24.65.

3,3'-(Sulfonylbis(2-(3-mercaptopropoxy)-5,1-phenylene))bis(propane-1-thiol) (**14b**). The product **14b** was obtained by the general procedure as stated above starting from **13b** (36.47 g, 51.00 mmol), conc. HCl (21.95 g), methanol (200 mL) and THF (50 mL). Purification by column chromatography with dichloromethane (R_f=0.66) as eluent afforded the desired product **14b** (20.6 g, 74%) as pale yellow oil. ^{1}H NMR (300 MHz, $CDCl_3$, δ [*ppm*]): 7.75 (dd, $^3J_{HH}$=8.6 Hz / $^4J_{HH}$=2.4 Hz, 2H, Ar***H***), 7.66 (d, $^4J_{HH}$= 2.4 Hz, 2H, Ar***H***), 6.90 (d, $^3J_{HH}$=8.6 Hz, 2H, Ar***H***), 4.13 (t, $^3J_{HH}$=5.9 Hz, 4H, OC***H₂***), 2.74 (m, 8H, C***H₂***SH and ArC***H₂***), 2.51 (m, 4H, C***H₂***SH), 2.17 – 2.05 (m, 4H, C***H₂***), 1.92 – 1.81 (m, 4H, C***H₂***), 1.39 (t, $^3J_{HH}$=8.1 Hz, 2H, S***H***), 1.38 (t, $^3J_{HH}$=7.8 Hz, 2H, S***H***); ^{13}C NMR (75 MHz, $CDCl_3$, δ [*ppm*]): 160.19 (Ar***C***), 133.76 (Ar***C***), 131.13 (Ar***C***), 129.05 (Ar***C***), 127.64 (Ar***C***), 111.08 (Ar***C***), 66.08 (ArO***C***H₂), 33.55 (***C***H₂), 33.06 (***C***H₂), 29.01 (Ar***C***H₂) 24.34 (***C***H₂S), 21.30 (***C***H₂S); FT-IR: *ν* = 3062 (w, ν_{ArH}), 2929 (w, ν_{CH2}), 2857 (w, ν_{CH2}), 2565 (w, ν_{SH}), 1592 (m, $\nu_{C=C}$), 1579 (m, $\nu_{C=C}$), 1490 (m, $\nu_{C=C}$),

1464 (m, δ_{CH2}), 1439 (m), 1412 (w), 1388 (w), 1307 (m), 1280 (m), 1249 (vs), 1180 (m), 1144 (s), 1116 (s), 1090 (vs), 1026 (m), 815 (m) cm^1; MS (EI) *m/z* (%): 547 (11) [M^+], 546 (37) [M^+], 439 (29), 438 (100), 398 (34), 364 (25), 331 (26), 330 (29), 317 (33), 305 (22), 304 (51), 250 (30), 197 (59), 181 (34), 166 (38), 149 (36), 134 (32), 133 (73), 75 (53), 47 (51), 41 (86); Anal. calc. for $C_{24}H_{34}O_4S_5$: C 52.71, H 6.27, S 29.32 found: C 52.74, H 6.28, S 29.00.

3,3',3''-(Ethane-1,1,1-triyltris(2-(3-mercaptopropoxy)benzene-5,1-diyl))tris(propane-1-thiol) (14c). The product **14c** was obtained by the general procedure as stated above starting from **13c** (26.59 g, 26.50 mmol), conc. HCl (17.11 g), methanol (200 mL) and THF (70 mL). Purification by column chromatography with dichloromethane/n-hexane 1/1 (R_f=0.57) as eluent afforded the desired product **14c** (16.0 g, 80%) as pale yellow oil. ^{1}H NMR (600 MHz, $CDCl_3$, δ [*ppm*]): 6.86 (s, 3H, Ar***H***), 6.80 (d, $^3J_{HH}$=8.6 Hz, 3H, Ar***H***), 6.70 (d, $^3J_{HH}$=8.6 Hz, 3H, Ar***H***), 4.05 (t, $^3J_{HH}$=5.8 Hz, 6H, OC$\boldsymbol{H_2}$), 2.75 (q, $^3J_{HH}$=7.4 Hz, 6H, C$\boldsymbol{H_2}$SH), 2.64 (t, $^3J_{HH}$=7.2 Hz, 6H, ArC$\boldsymbol{H_2}$), 2.46 (t, $^3J_{HH}$=7.3 Hz, 6H, C$\boldsymbol{H_2}$SH), 2.12 – 2.05 (m, 9H, C$\boldsymbol{H_2}$ and C(C$\boldsymbol{H_3}$)), 1.86 – 1.78 (m, 6H, C$\boldsymbol{H_2}$), 1.41 (t, $^3J_{HH}$=8.0 Hz, 3H, S***H***), 1.33 (t, $^3J_{HH}$=8.0 Hz, 3H, S***H***); ^{13}C NMR (150 MHz, $CDCl_3$, δ [*ppm*]): 154.67 (Ar***C***), 141.72 (Ar***C***), 130.74 (Ar***C***), 128.83 (Ar***C***), 127.24 (Ar***C***), 110.29 (Ar***C***), 65.54 (O***C***H_2), 50.70 (***C***(CH_3)), 34.29 (***C***H_2), 33.62 (***C***H_2), 30.92 (C(***C***H_3)), 29.36 (***C***H_2), 24.39 (***C***H_2), 21.61 (***C***H_2); FT-IR: ν = 3050 (w), 3028 (w, ν_{CH}), 2961 (w, ν_{CH3}), 2923 (m, ν_{CH2}), 2865 (w, ν_{CH2}), 2558 (w, ν_{SH}), 1605 (w, $\nu_{C=C}$), 1581 (w, $\nu_{C=C}$), 1497 (vs, $\nu_{C=C}$), 1467 (m, δ_{CH2}), 1433 (w), 1386 (w, δ_{CH3}), 1360 (w, δ_{CH3}), 1348 (w), 1293 (m), 1243 (vs), 1149 (m), 1117 (s), 1032 (m), 813 (m) cm^{-1}; MS (EI) *m/z* (%): 738 (11) [M-CH_3^+], 737 (33) [M-CH_3^+], 736 (38) [M-CH_3^+], 735 (100) [M-CH_3^+], 662 (11), 661 (30), 513 (20), 361 (13), 193 (19), 159 (28), 75 (14), 47 (12), 41 (17); Anal. calc. for $C_{38}H_{54}O_3S_6$: C 60.76, H 7.25, S 25.61 found: C 60.96, H 7.22, S 25.42.

3,3',3'',3'''-(Propane-2,2-diylbis(2-(3-mercaptopropoxy)benzene-5,3,1-triyl))tetrakis-(propane-1-thiol) (17). The product **17** was obtained by the general procedure as stated

above starting from **16** (20.36 g, 22.00 mmol), conc. HCl (14.21 g), methanol (200 mL) and THF (50 mL). The product **17** (13.9 g, 94%) was obtained as pale yellow oil and used without further purification. ^{1}H NMR (300 MHz, $CDCl_3$, δ [*ppm*]): 6.75 (s, 4H, Ar***H***), 3.80 (t, $^{3}J_{HH}$=6.0 Hz, 4H, OC$\boldsymbol{H_2}$), 2.78 – 2.69 (m, 4H, C$\boldsymbol{H_2}$S), 2.64 – 2.55 (m, 8H, ArC$\boldsymbol{H_2}$), 2.47 – 2.38 (m, 8H, C$\boldsymbol{H_2}$S), 2.08 – 1.97 (m, 4H, C$\boldsymbol{H_2}$), 1.86 – 1.73 (m, 8H, C$\boldsymbol{H_2}$), 1.53 (s, 6H, C(C$\boldsymbol{H_3}$)$_2$), 1.37 (t, $^{3}J_{HH}$=8.0 Hz, 2H, S***H***), 1,31 (t, $^{3}J_{HH}$=7.9 Hz, 4H, S***H***); ^{13}C NMR (75 MHz, $CDCl_3$, δ [*ppm*]): 153.07 (Ar***C***), 146.38 (Ar***C***), 133.56 (Ar***C***), 126.70 (Ar***C***), 71.34 (O***C***H_2), 42.17 (***C***$(CH_3)_2$), 35.00 (***C***H_2S), 34.59 (***C***H_2S), 31.06 (**C**(***C***H_3)$_2$), 29.03 (Ar***C***H_2), 24.44 (***C***H_2), 21.52 (***C***H_2); FT-IR: v = 3041 (w, ν_{CH}), 2961 (m, ν_{CH3}), 2927 (m, ν_{CH2}), 2858 (w, ν_{CH2}), 2559 (w, ν_{SH}), 1598 (w, $\nu_{C=C}$), 1469 (s, δ_{CH2}), 1426 (m), 1381 (w, δ_{CH3}), 1360 (w, δ_{CH3}), 1277 (m), 1262 (m), 1224 (m), 1176 (s), 1103 (m), 1021 (m) cm^{-1}; MS (EI) *m*/*z* (%): 673 (9) [M$^+$], 672 (21) [M$^+$], 657 (12), 598 (40), 584 (20), 583 (60), 524 (28), 511 (21), 510 (30), 509 (100), 475 (28), 435 (29), 401 (21), 316 (30), 283 (66), 281 (22), 269 (67), 249 (40), 235 (29), 221 (32), 209 (26), 207 (20) 189 (22), 187 (142) 175 (55), 173 (37), 161 (21), 159 (34), 147 (58), 145 (23), 87 (41), 75 (34), 47 (30), 41 (41); Anal. calc. for $C_{33}H_{52}O_2S_6$: C 58.88, H 7.91 S 28.58 found: C 58.84, H 7.77, S 28.24.

G. Preparation and testing of composites

The experimental resins or composites were mixed in an LPM 0.1 SP kneading machine (Linden, Marienheide, Germany). Flexural strength specimens (2 mm x 2 mm x 25 mm) were obtained by irradiating the composites with a visible light source (Spectramat, Ivoclar Vivadent AG, Schaan, Liechtenstein) for 3 min per side of specimen. In this case, light of 380-500 nm wavelength and an intensity of 150 mW/cm^2 was used. The mechanical properties were determined according to the relevant standard EN ISO 4049:2009[7]. The flexural strength and flexural modulus of elasticity were determined using a BZ2.5/TS1S

universal testing machine (Zwick GmbH & Co. KG, Ulm, Germany) after storing the cured specimens at ambient temperature for 24 h or after immersing the cured specimens in water for 24 h at 37 °C. The exothermic time and temperature were measured using a Heliolux DLX (600 mW/cm^2, Ivoclar Vivadent AG).

The conversion was measured using a Spectrum One FT-IR photometer (Perkin Elmer) equipped with Golden Gate Single Reflection Diamond ATR (Specac Ltd, Orpington, UK) in absorbance mode. The peak height of the –S-H peak (2570 cm^{-1}) and the =C-H peak (3082 cm^{-1}) respectively were compared to the >C=O peak (1680 cm^{-1}) as internal standard for the uncured sample and the cured sample (2 x 3 min, Spectramat, Ivoclar Vivadent AG). The degree of conversion can be expressed by the following relation:

$$\text{conversion}(\%) = (H_0-H_t)\times 100/H_0$$

where H_0 is the initial peak height before irradiation and H_t is the peak height of the thiol bonds or the double bonds at time t. For each sample, real-time IR spectroscopy was repeated three times.

The shrinkage force was measured based on a method described by Watts et al.[8] with a universal BZ2.5/TS1S universal testing machine (Zwick) in static mode. The sample was placed between a glass slide and a sand blasted steel rod (diameter 10 mm) mounted to the measuring cell. Steel and glass surfaces were treated with Monobond Plus® (Ivoclar Vivadent AG). The force occurring during irradiation with a dental LED light source (10 s, BluePhase 20i, Ivoclar Vivadent AG) of a 0.8 mm thick sample was recorded for additional 8 min. For each sample, shrinkage force measurements were repeated six times.

^{1}H and ^{13}C NMR spectra

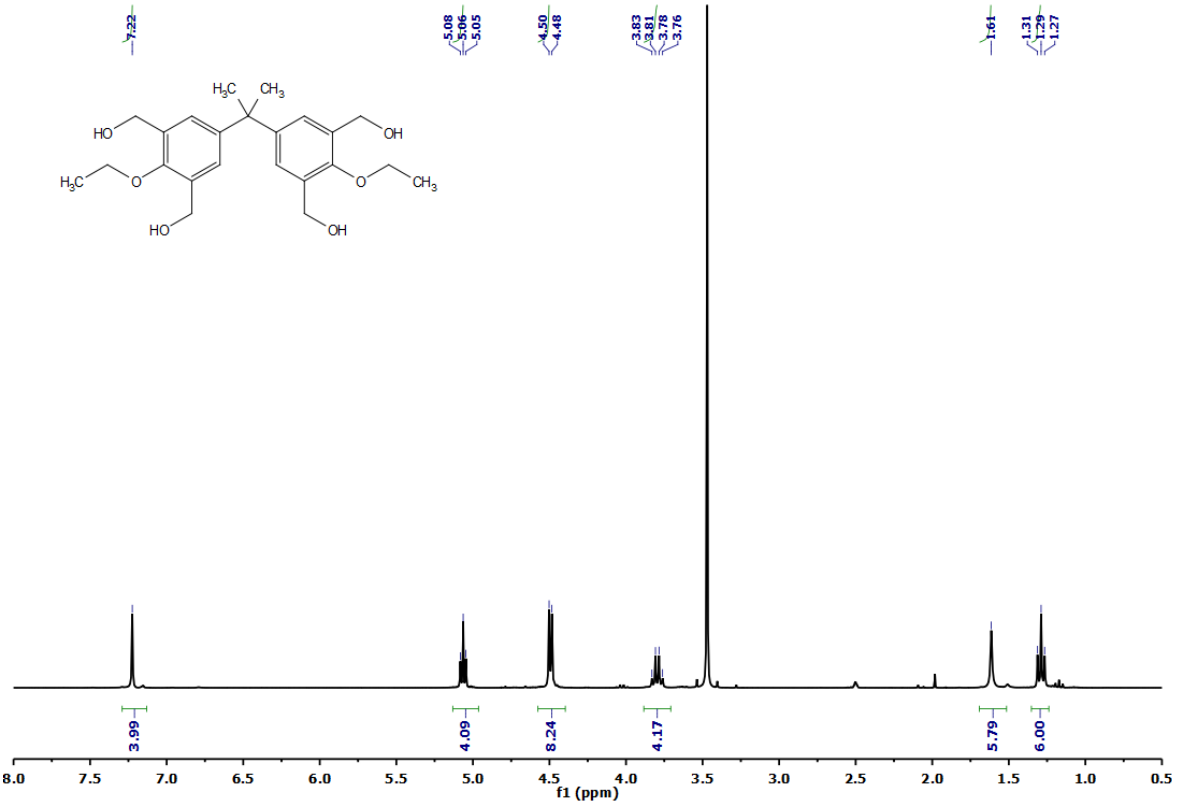

Figure S1. **^{1}H NMR spectrum of 2,2-Bis(3,5-bis(hydroxymethyl)-4-ethoxyphenyl)propane (5) in DMSO-d_6 (300 MHz, rt).**

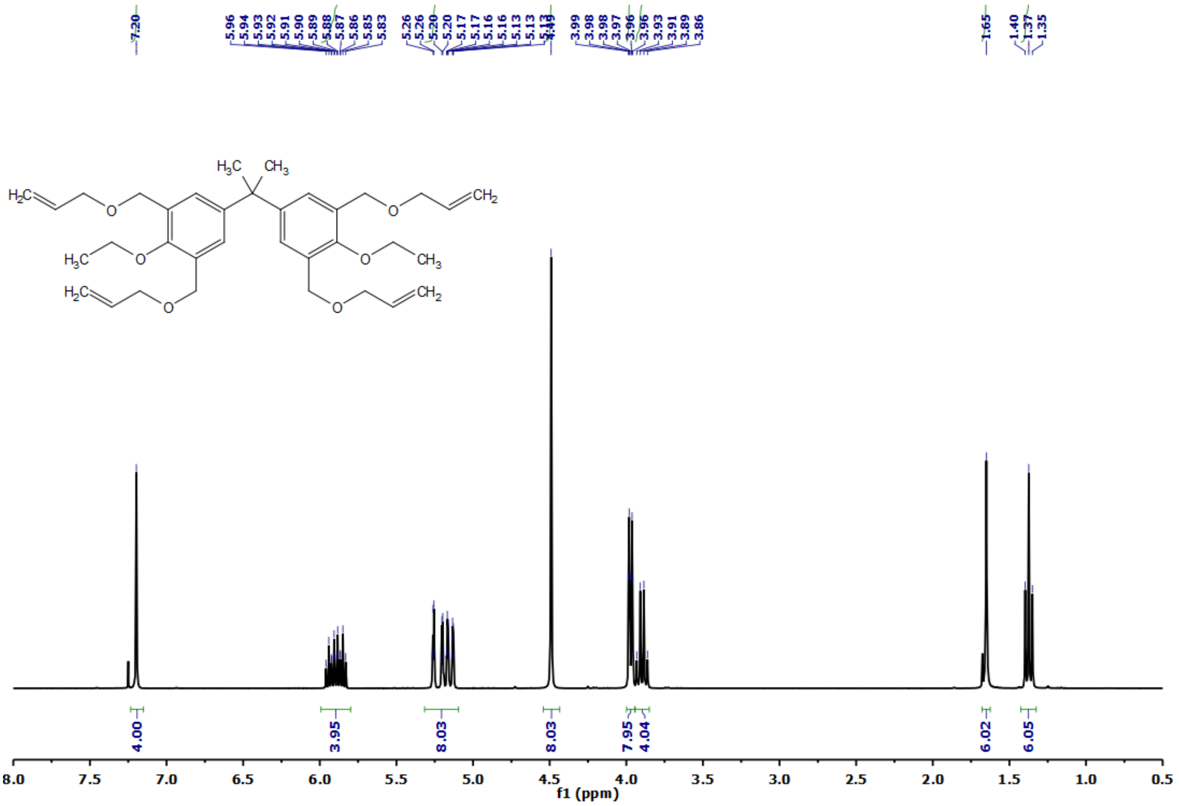

Figure S2. **^{1}H NMR spectrum of 5,5'-(Propane-2,2-diyl)bis(1,3-bis(allyloxy)methyl)-2-ethoxybenzene (7a) in $CDCl_3$ (300 MHz, rt).**

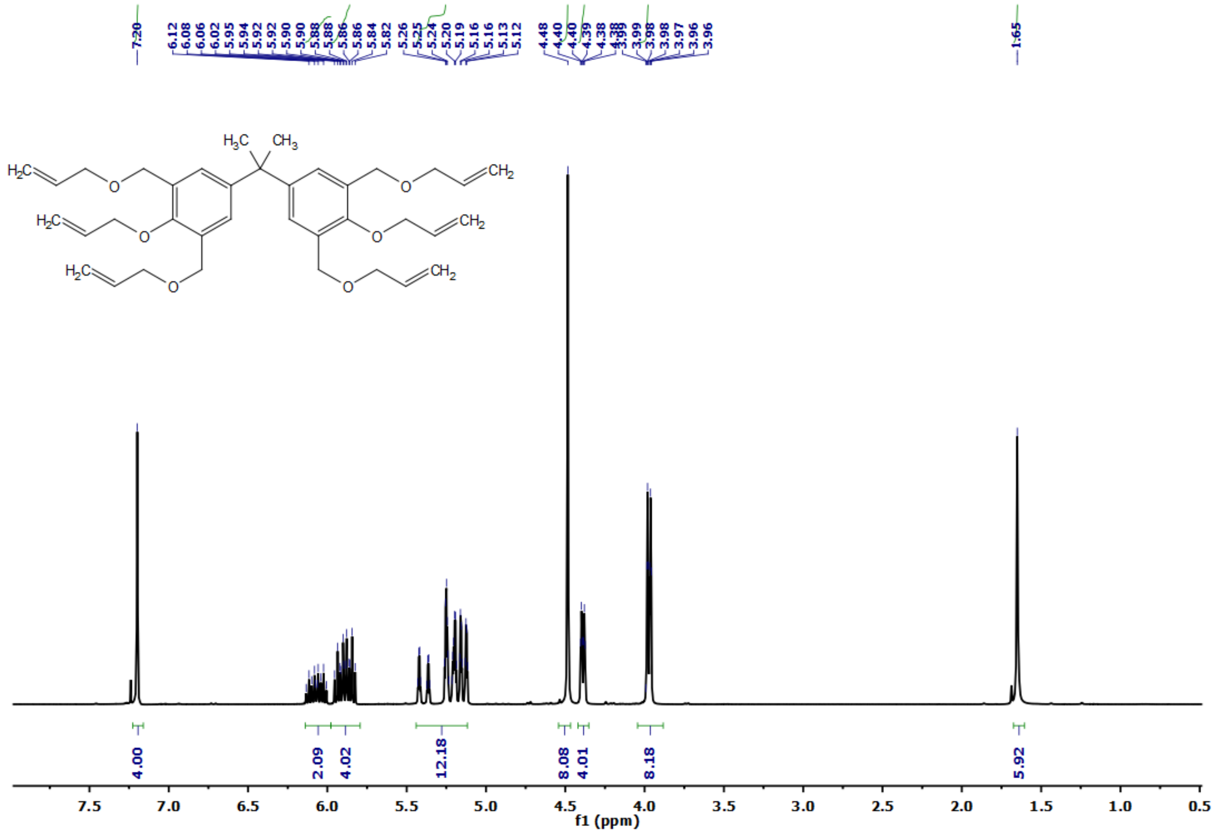

Figure S3. ^{1}**H NMR spectrum of 5,5'-(Propane-2,2-diyl)bis(2-(allyloxy)-1,3-bis((allyloxy)mehtyl)benzene (7b) in CDCl$_3$ (300 MHz, rt).**

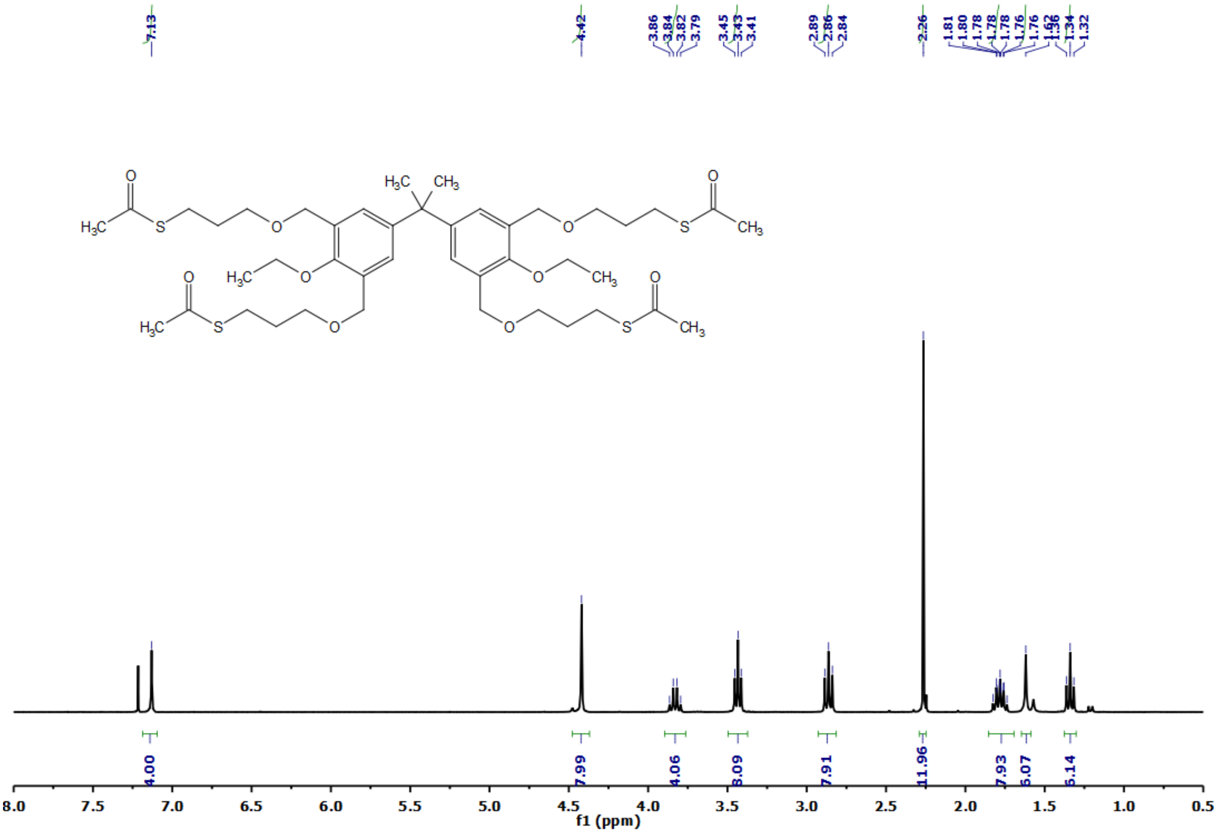

Figure S4. ^{1}**H NMR spectrum of S,S',S'',S'''-((Propane-2,2-diylbis(2-ethoxybenzene-5,3,1-triyl)tetrakis(methylene)-tetrakis-(oxy))tetrakis(propane-3,1-diyl)) tetraethanethioate (9a) in CDCl$_3$ (300 MHz, rt).**

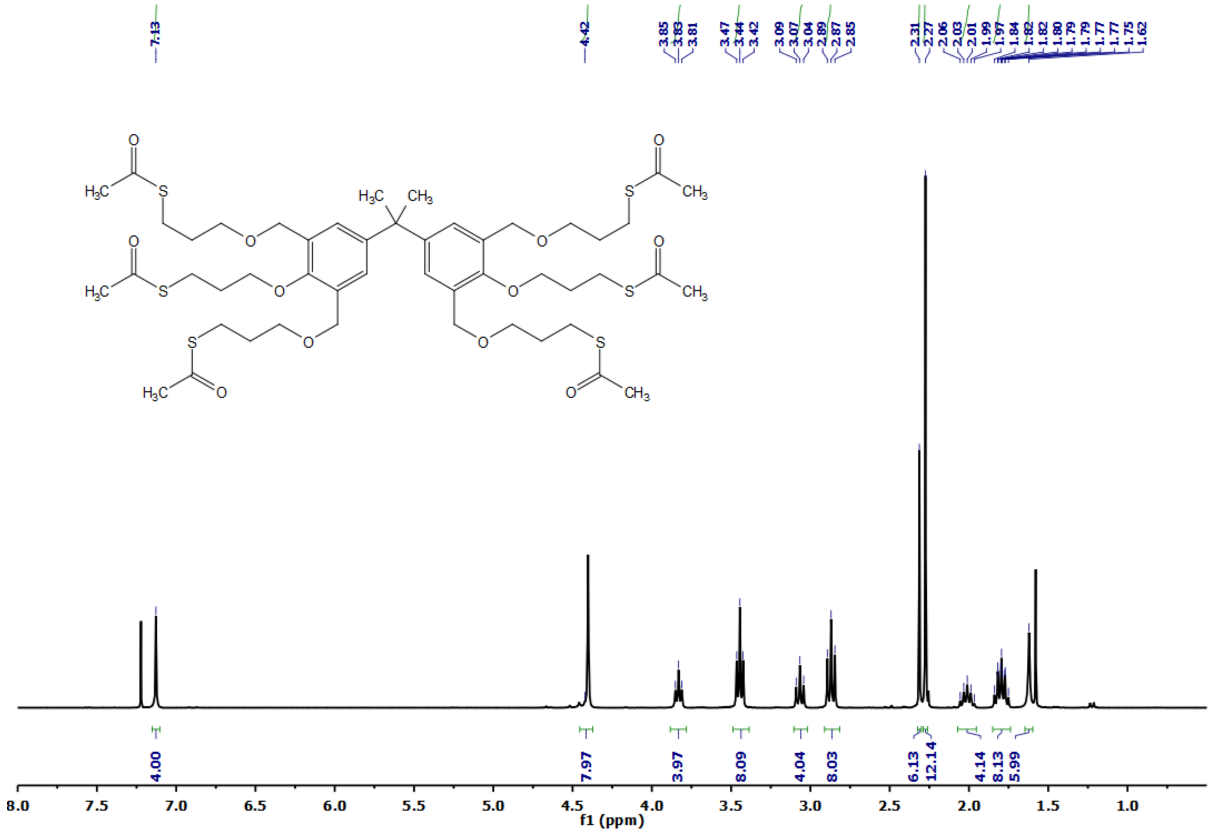

Figure S5. ¹H NMR spectrum of S,S',S'',S'''-((((Propane-2,2-diylbis(2-(3-(acetylthio)propoxy)benzene-5,3,1-triyl))-tetrakis(methylene))tetrakis(oxy))tetrakis(propane-3,1-diyl)) tetraethanethioate (9b) in $CDCl_3$ (300 MHz, rt).

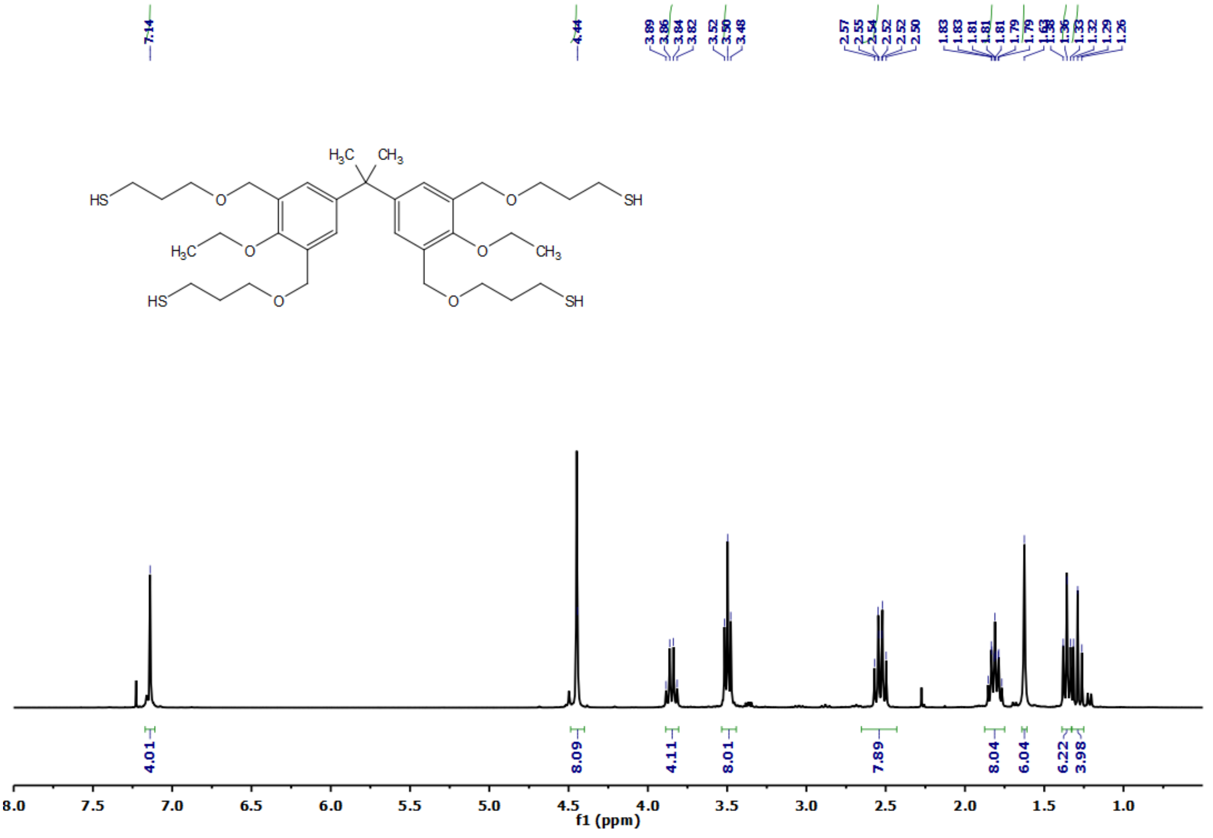

Figure S6. ¹H NMR spectrum of 3,3',3'',3'''-(((Propane-2,2-diylbis(2-ethoxybenzene-5,3,1-triyl))tetrakis-(methylene))tetrakis(oxy))tetrakis(propane-1-thiol) (10a) in $CDCl_3$ (300 MHz, rt).

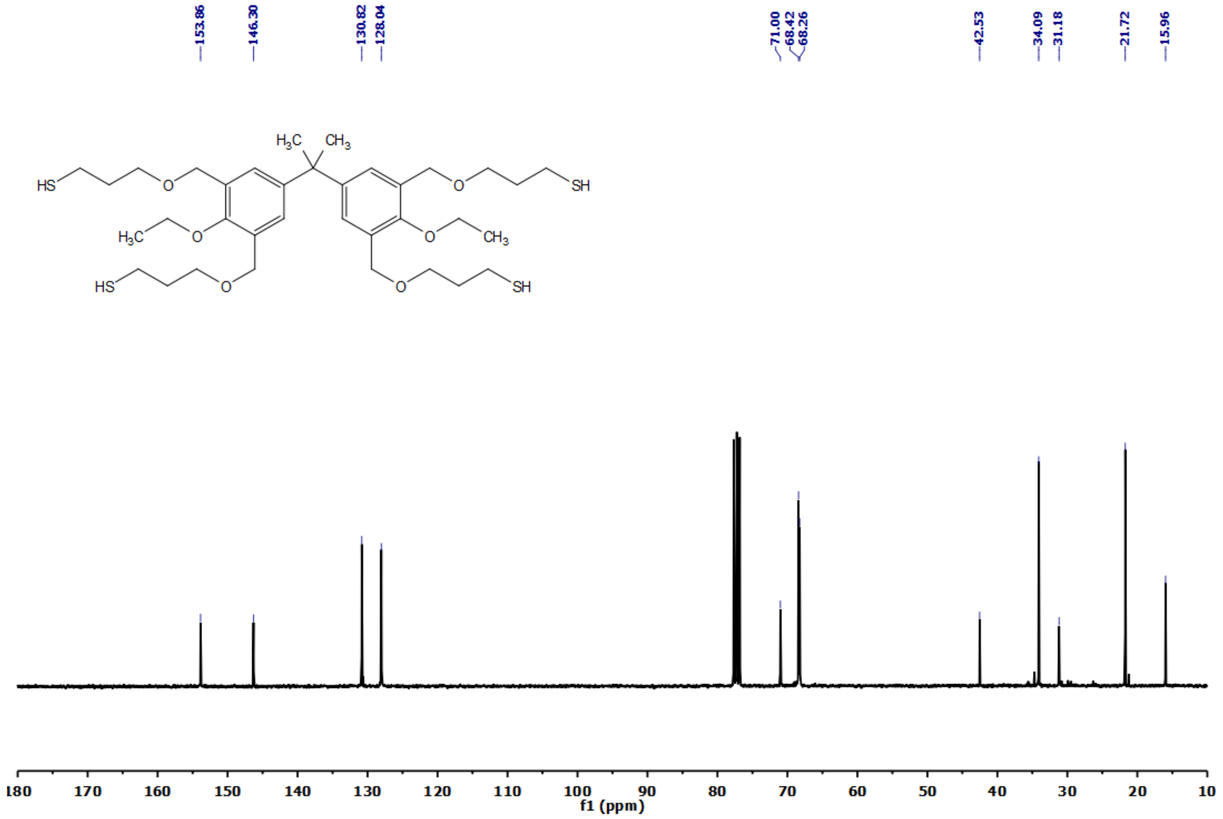

Figure S7. **^{13}C NMR spectrum of 3,3',3'',3'''-(((Propane-2,2-diylbis(2-ethoxybenzene-5,3,1-triyl))tetrakis-(methylene))tetrakis(oxy))tetrakis(propane-1-thiol) (10a) in $CDCl_3$ (75 MHz, rt).**

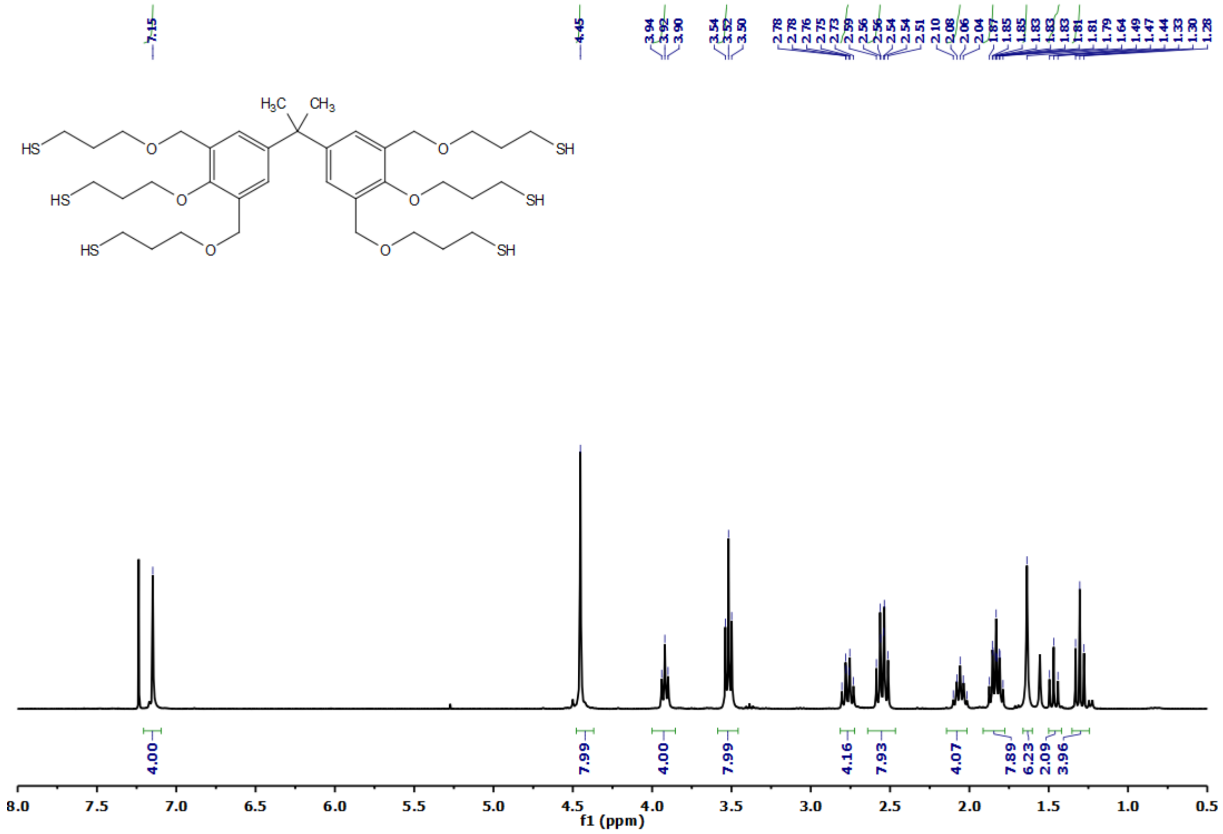

Figure S8. **^{1}H NMR spectrum of 3,3',3'',3'''-((Propane-2,2-diylbis(2-(3-mercaptopropoxy)benzene-5,3,1-triyl)-tetrakis(methylene))tetrakis(oxy))tetrakis(propane-1-thiol) (10b) in $CDCl_3$ (300 MHz, rt).**

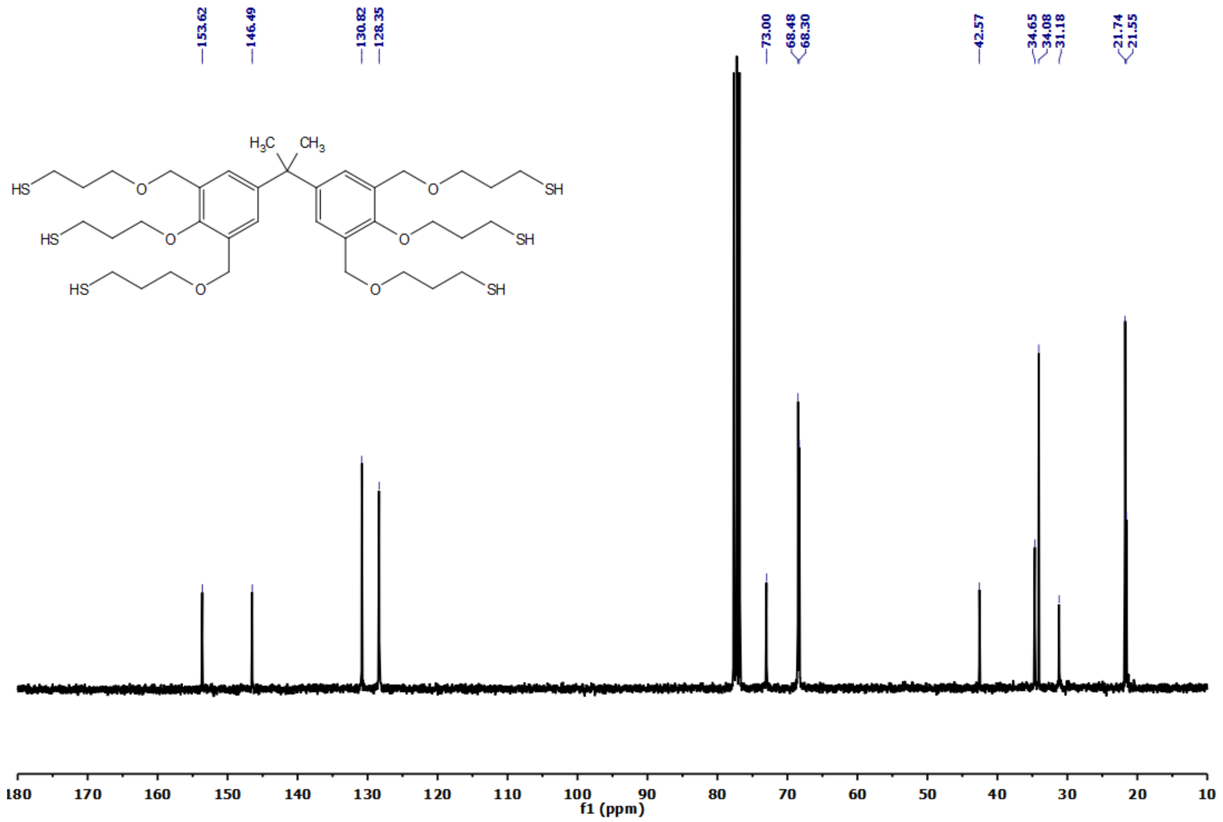

Figure S9. [13]**C NMR spectrum of 3,3',3'',3'''-((Propane-2,2-diylbis(2-(3-mercaptopropoxy)benzene-5,3,1-triyl)-tetrakis(methylene))tetrakis(oxy))tetrakis(propane-1-thiol) (10b) in $CDCl_3$ (75 MHz, rt).**

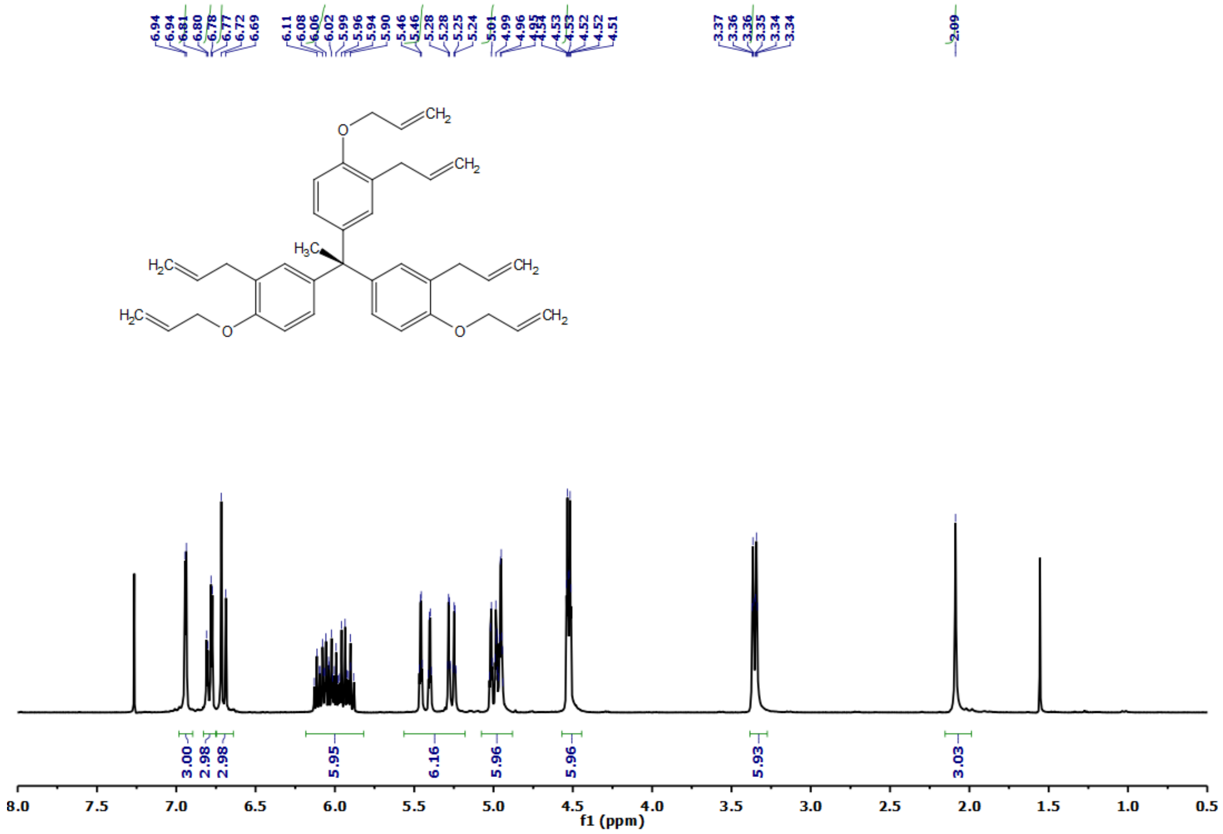

Figure S10. [1]**H NMR spectrum of 4,4',4''-(Ethane-1,1,1-triyl)tris(2-allyl-1-(allyloxy)benzene) (12c) in $CDCl_3$ (300 MHz, rt).**

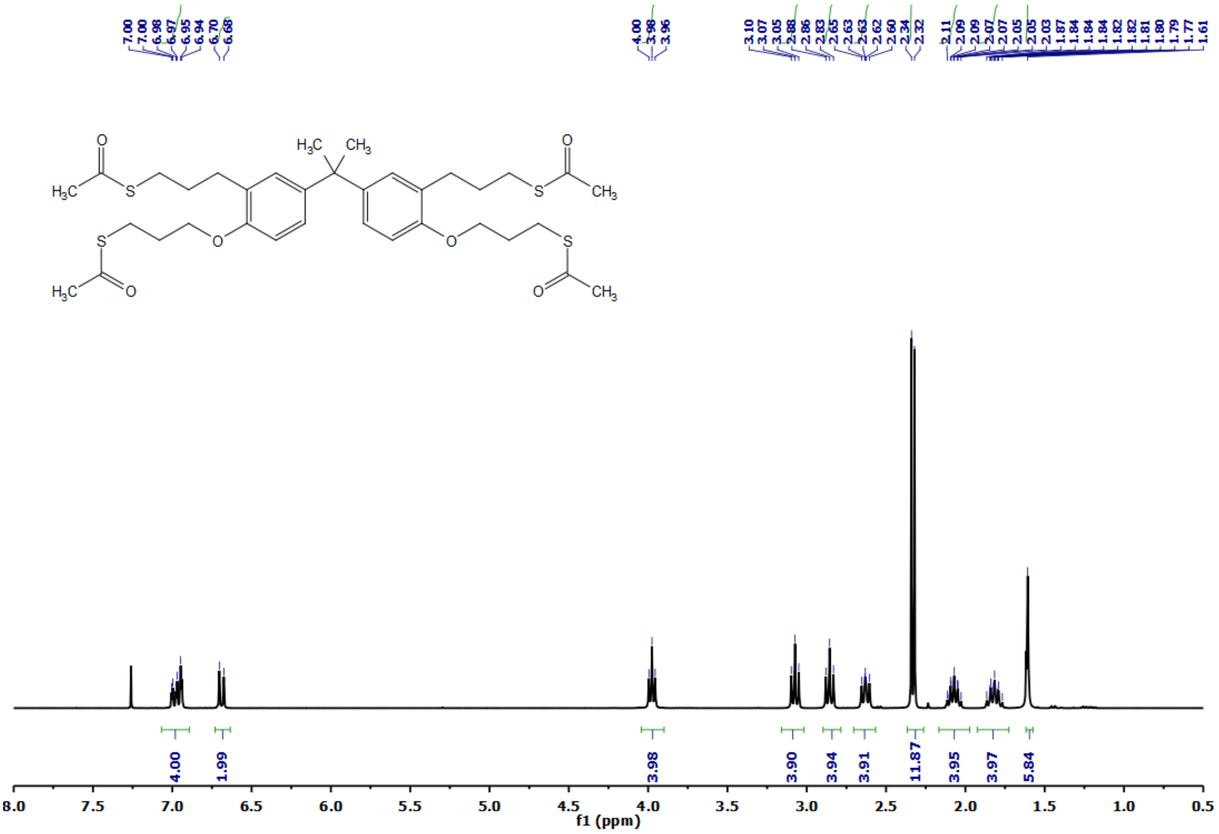

Figure S11. **^{1}H NMR spectrum of S,S'-((Propane-2,2-diylbis(2-(3-(acetylthio)propoxy)-5,1-phenylene))bis(propane-3,1-diyl)) diethanethioate (13a) in $CDCl_3$ (300 MHz, rt).**

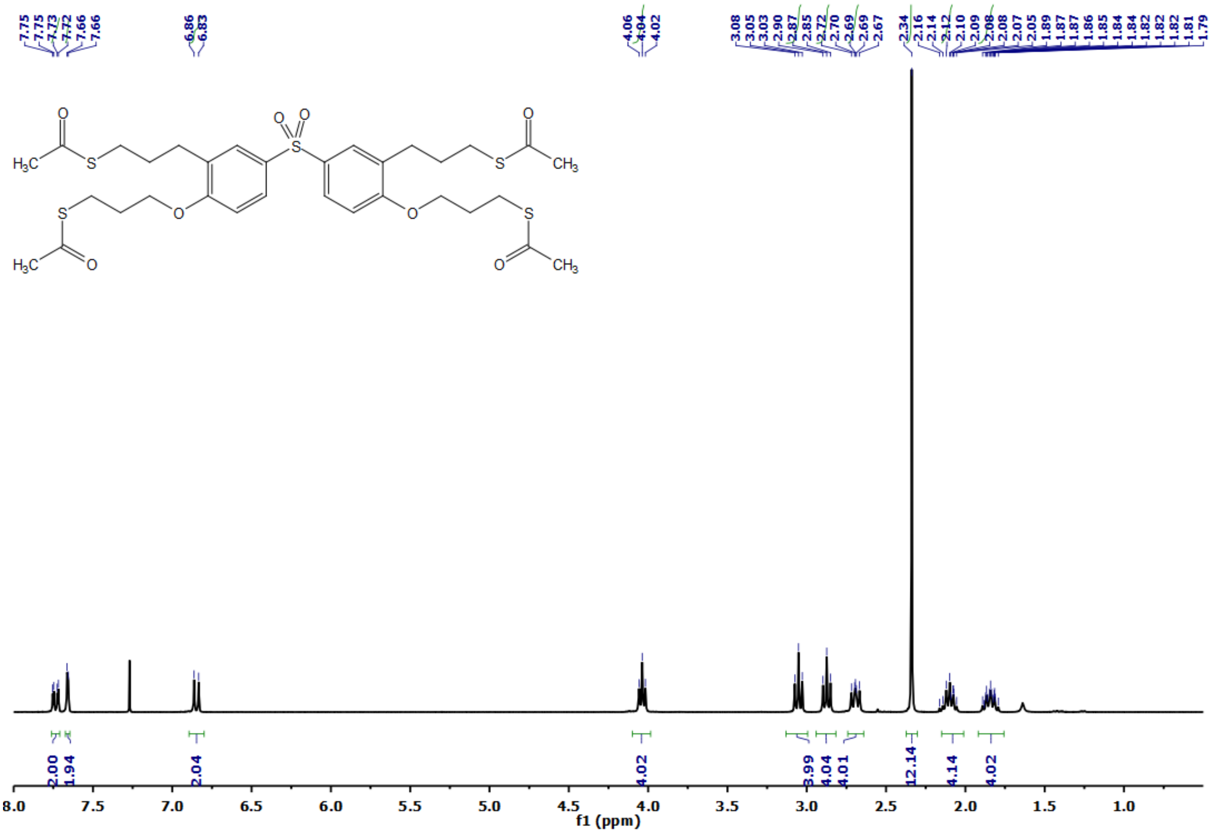

Figure S12. **^{1}H NMR spectrum of S,S'-((Sulfonylbis(2-(3-(acetylthio)propoxy)-5,1-phenylene))bis(propane-3,1-diyl)) diethanethioate (13b) in $CDCl_3$ (300 MHz, rt).**

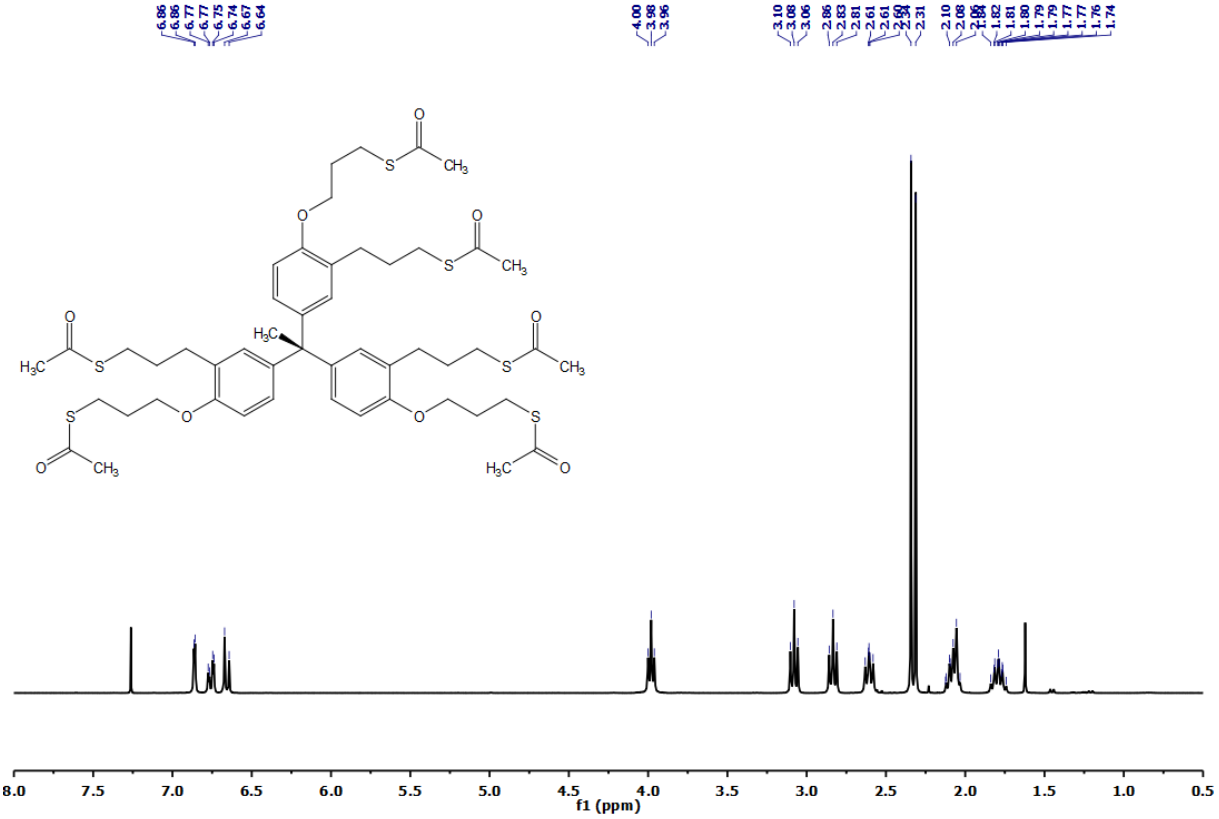

Figure S13. [1]**H NMR spectrum of S,S',S''-((Ethane-1,1,1-triyltris(2-(3-(acetylthio)propoxy)benzene-5,1-diyl))tris(propane-3,1-diyl)) triethanethioate (13c) in $CDCl_3$ (300 MHz, rt).**

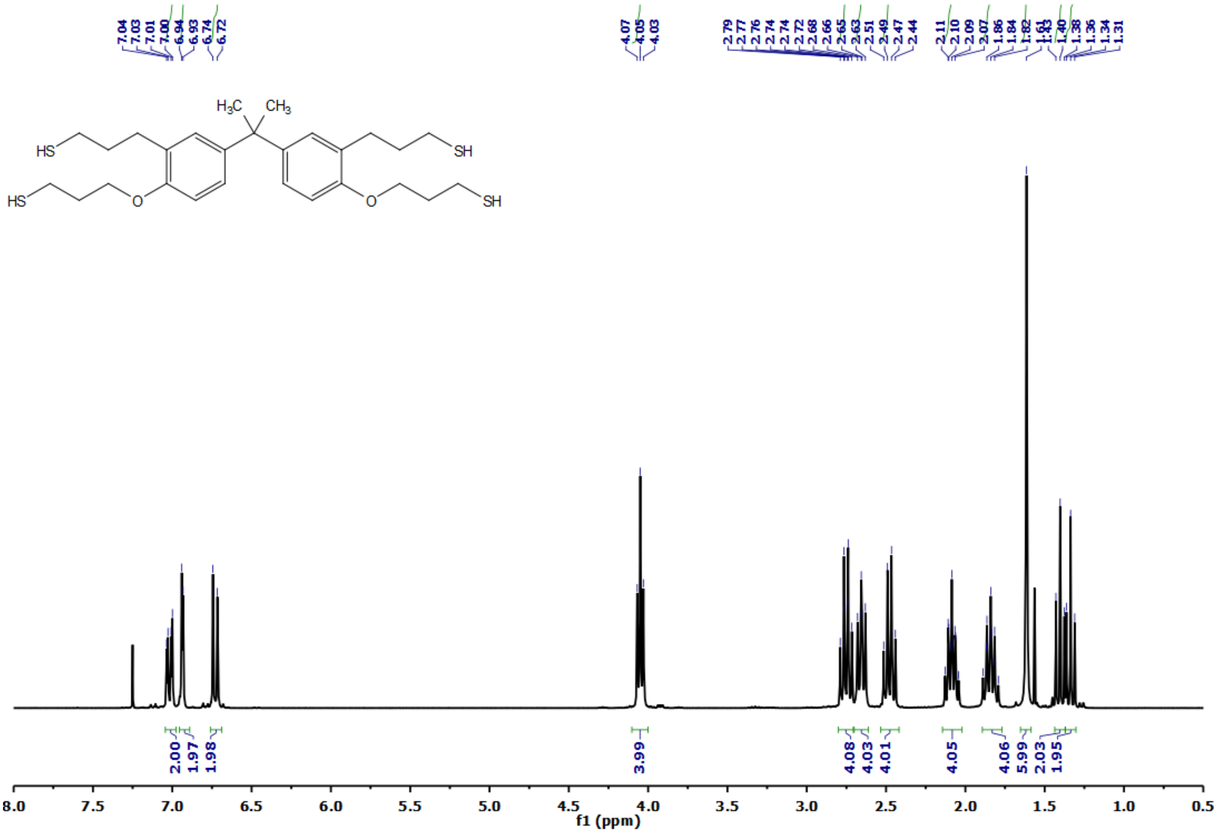

Figure S14. [1]**H NMR spectrum of 3,3'-(Propane-2,2-diylbis(2-(3-mercaptopropoxy)-5,1-phenylene))bis(propane-1-thiol) (14a) in $CDCl_3$ (300 MHz, rt).**

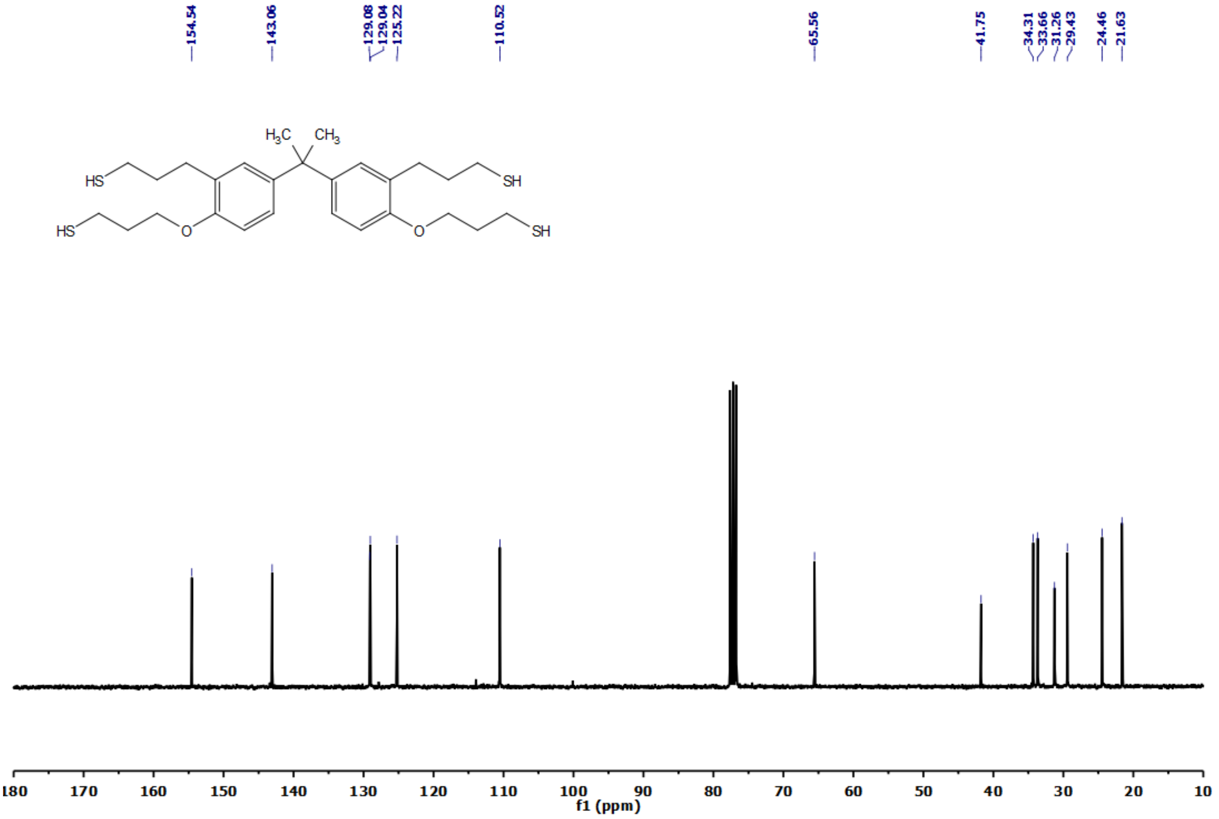

Figure S15. ^{13}**C NMR spectrum of 3,3'-(Propane-2,2-diylbis(2-(3-mercaptopropoxy)-5,1-phenylene))bis(propane-1-thiol) (14a) in CDCl$_3$ (75 MHz, rt).**

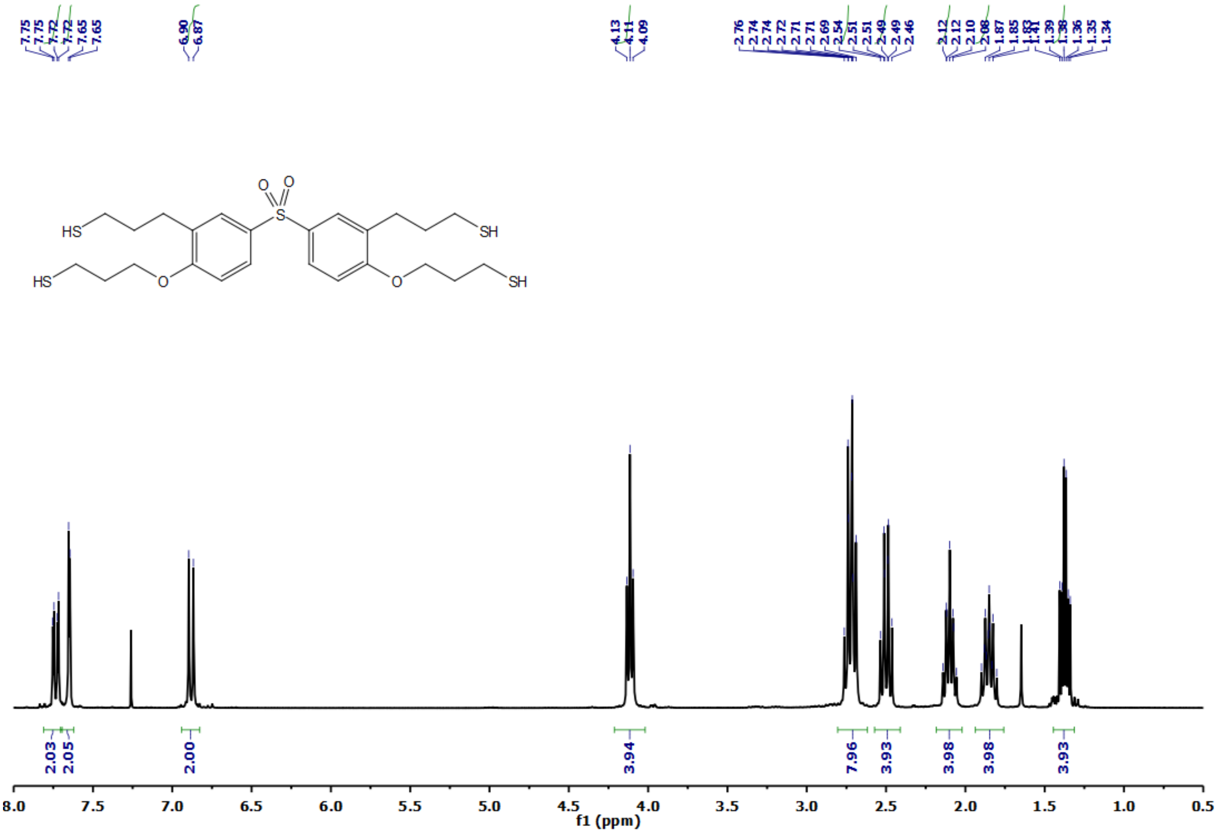

Figure S16. ^{1}**H NMR spectrum of 3,3'-(Sulfonylbis(2-(3-mercaptopropoxy)-5,1-phenylene))bis(propane-1-thiol) (14b) in CDCl$_3$ (300 MHz, rt).**

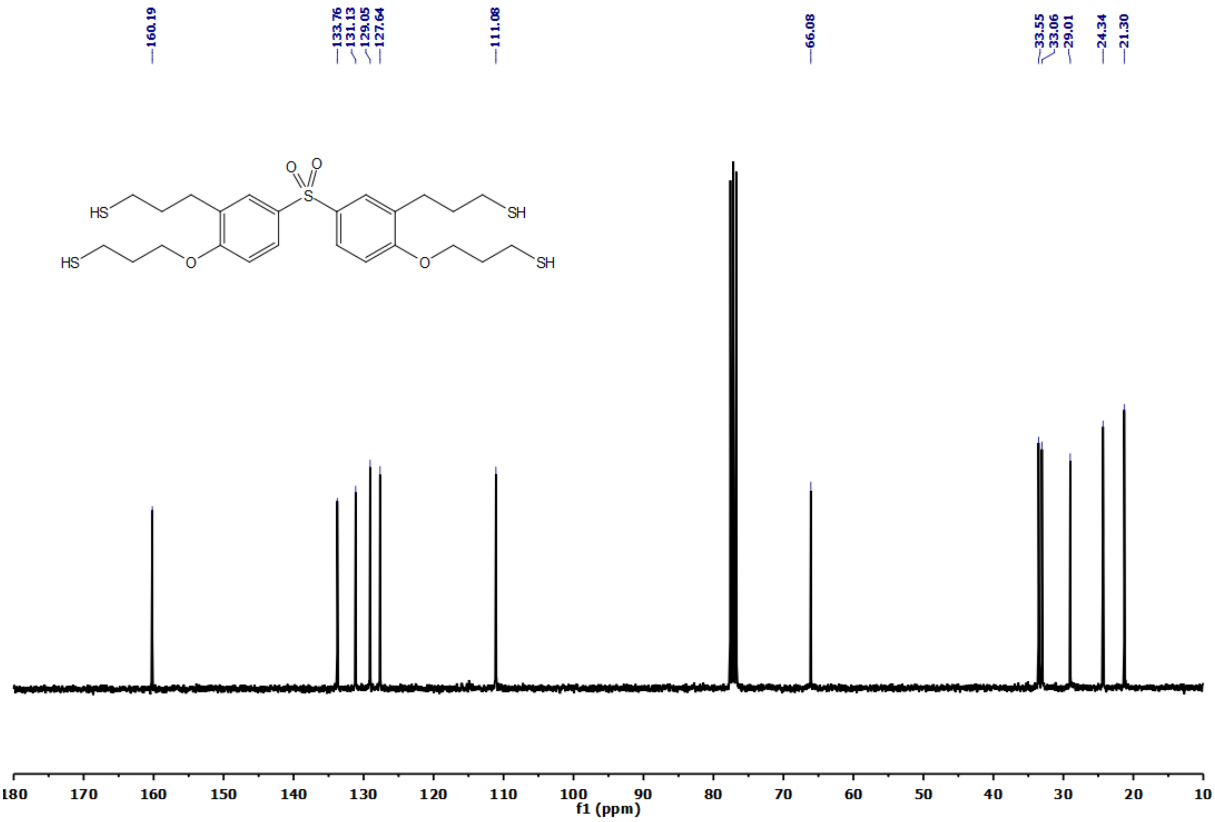

Figure S17. ^{13}**C NMR spectrum of 3,3'-(Sulfonylbis(2-(3-mercaptopropoxy)-5,1-phenylene))bis(propane-1-thiol) (14b) in $CDCl_3$ (300 MHz, rt).**

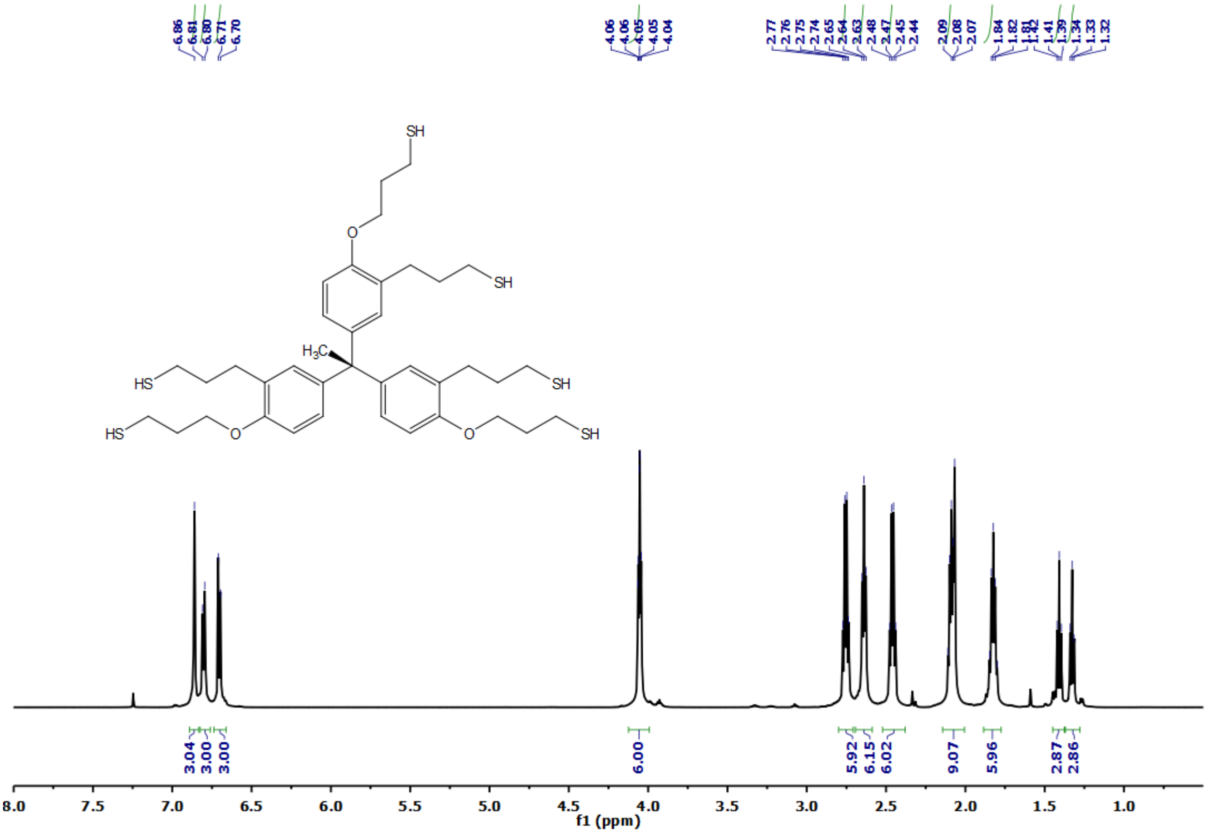

Figure S18. ^{1}**H NMR spectrum of 3,3',3''-(Ethane-1,1,1-triyltris(2-(3-mercaptopropoxy)benzene-5,1-diyl))tris-(propane-1-thiol) (14c) in $CDCl_3$ (600 MHz, rt).**

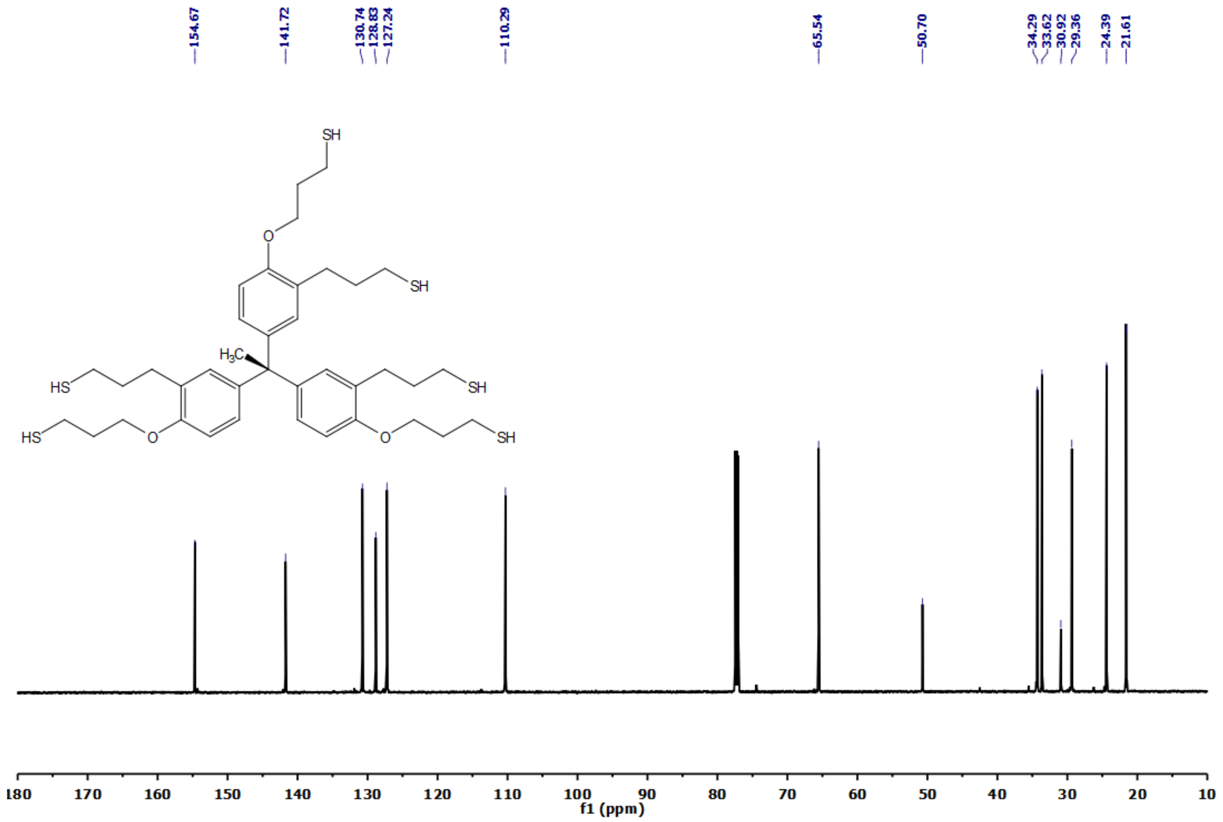

Figure S19. ^{13}C NMR spectrum of 3,3',3''-(Ethane-1,1,1-triyltris(2-(3-mercaptopropoxy)benzene-5,1-diyl))tris-(propane-1-thiol) (14c) in $CDCl_3$ (150 MHz, rt).

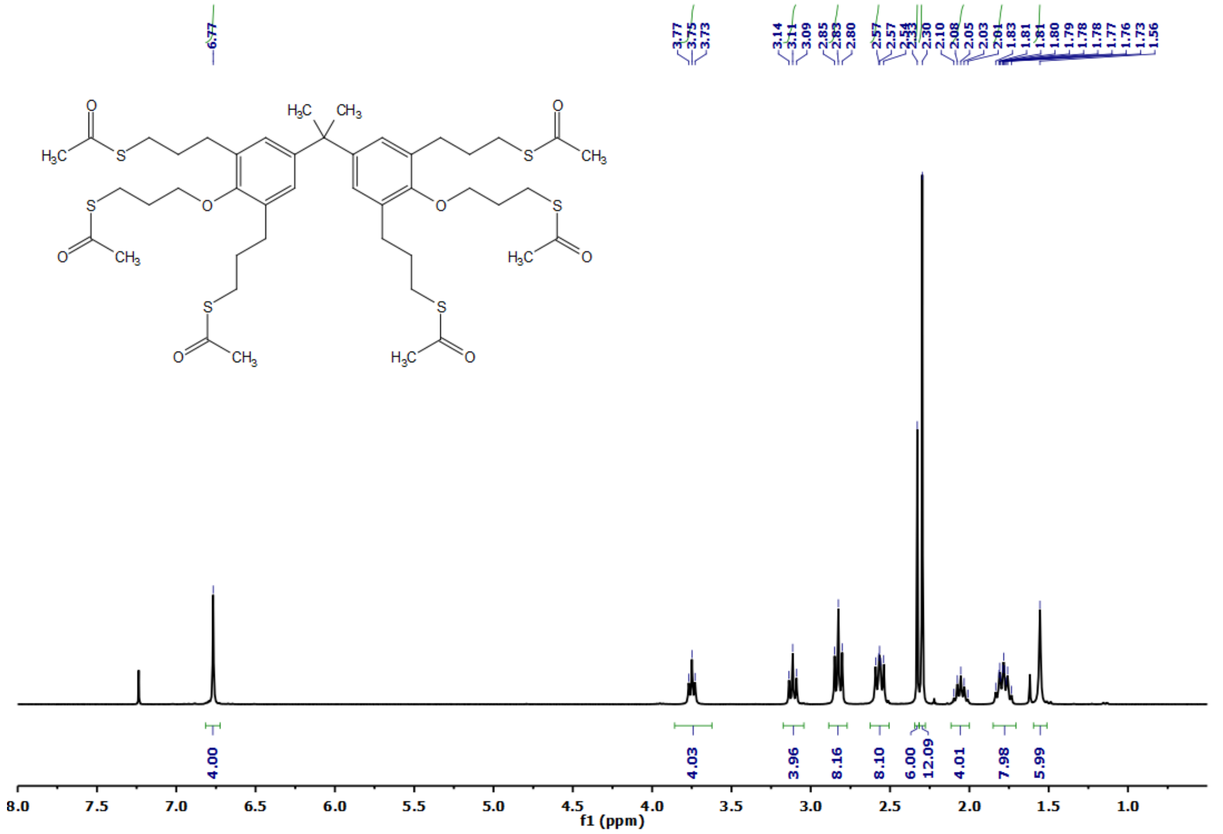

Figure S20. ^{1}H NMR spectrum of S,S',S'',S'''-(Propane-2,2-diylbis(2-(3-(acetylthio)propoxy)benzene-5,3,1-triyl))tetrakis(propane-3,1-diyl)) tetraethanethioate (16) in $CDCl_3$ (300 MHz, rt).

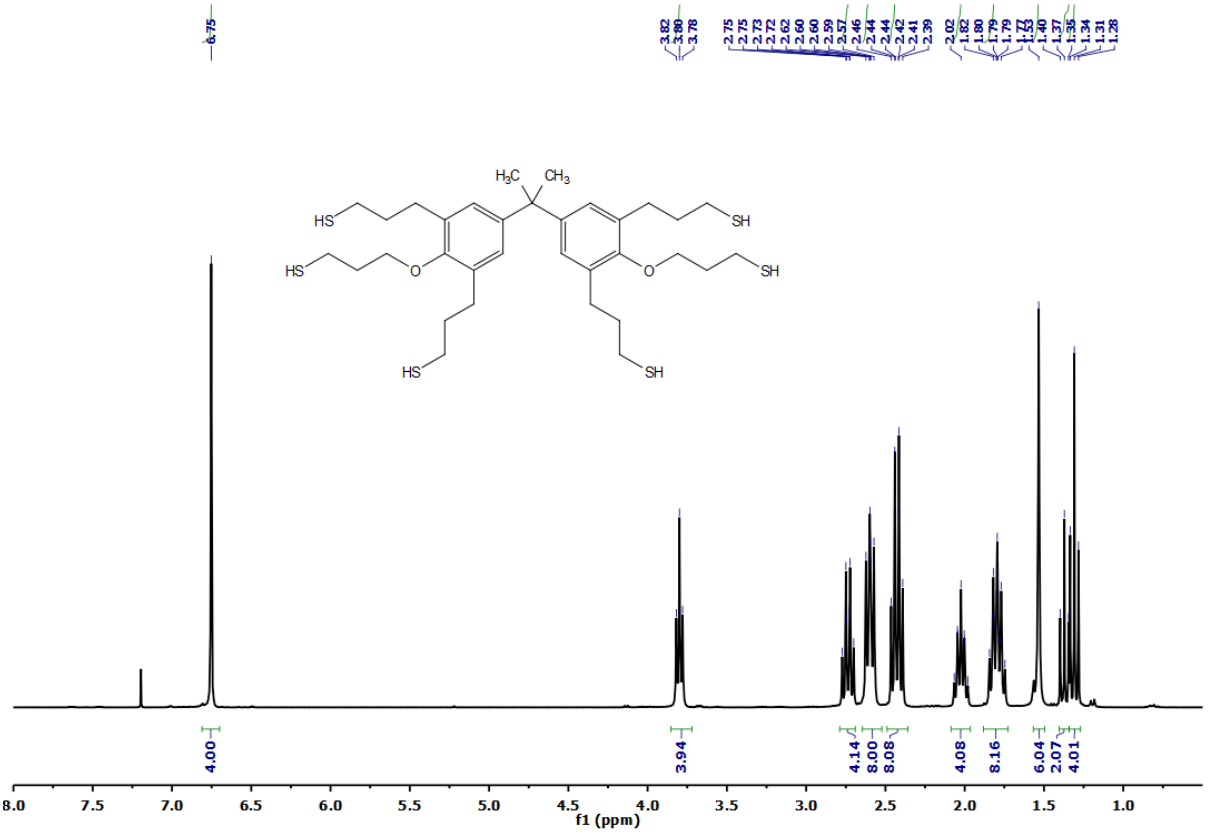

Figure S21. ^{1}H NMR spectrum of 3,3',3'',3'''-(Propane-2,2-diylbis(2-(3-mercaptopropoxy)benzene-5,3,1-triyl))tetrakis(propane-1-thiol) (17) in $CDCl_3$ (300 MHz, rt).

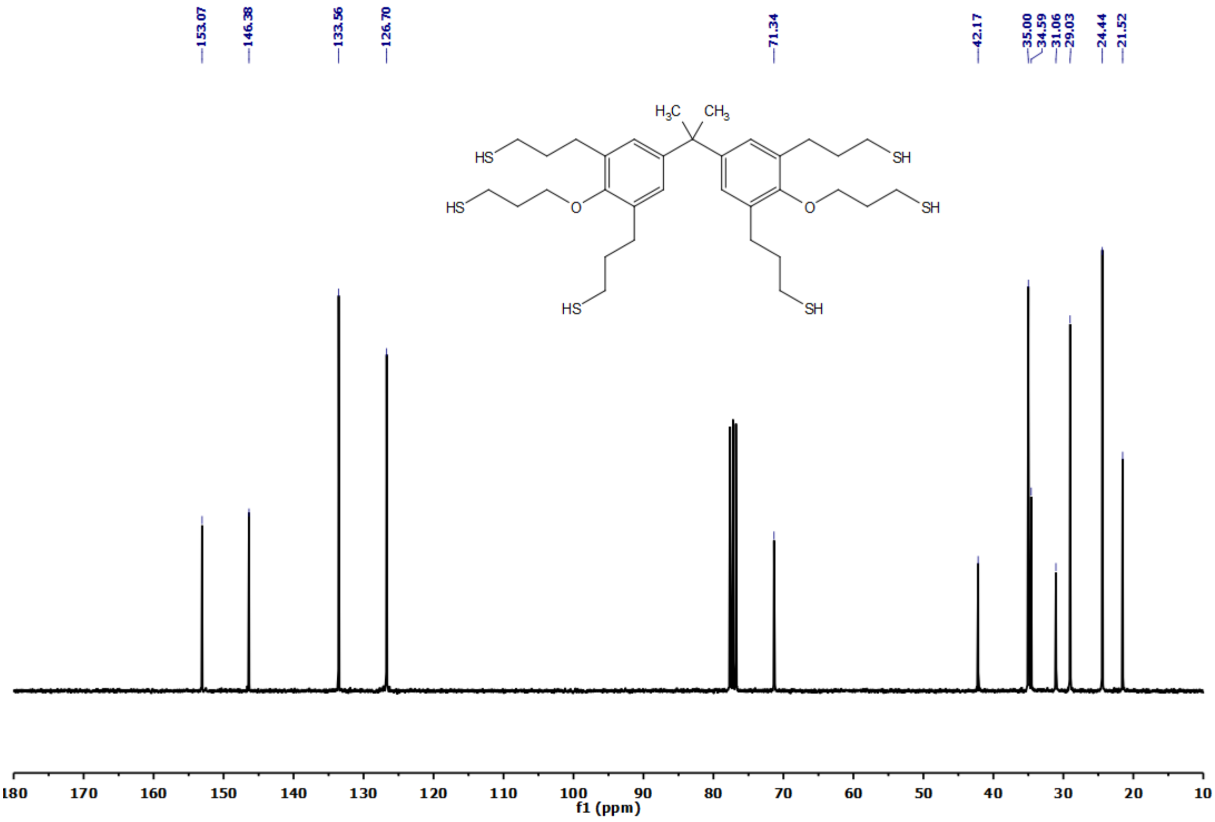

Figure S22. ^{13}C NMR spectrum of 3,3',3'',3'''-(Propane-2,2-diylbis(2-(3-mercaptopropoxy)benzene-5,3,1-triyl))tetrakis(propane-1-thiol) (17) in $CDCl_3$ (75 MHz, rt).

REFERENCES

1. Junek, H.; Pampouchidis, G.; Rauch-Puntigam, H. *Monatsh. Chem.* **1973**, *104*, 1077-1089.
2. Sorrell, T. N.; Yuan, H. *J. Org. Chem.* **1997**, *62*, 1899-1902.
3. Shinmoto, M. Phenolic compound, preparation thereof and recording material employing same. US4596997A, June 24, 1986.
4. Abraham, M.; Hamerton, I.; Rose, J.; Grate, J. *J. Chem. Soc., Perkin Trans. 2***1991**, *1417-1423*.
5. Jethmalani, J.; Dreher, A. W.; Abdel-Sadek, G.; Chomyn, J.; Li, J.; Qaddoura, M. Monomers and polymers for optical elements. US20060052547A1, March 9, 2006.
6. Herr, D. E.; Hallock, J. S. Photocurable composition based on acid functional primary resinous mercaptans. WO9858294A1, December 23, 1998.
7. Dentistry - Polymer-based filling resorative and luting materials; International standard ISO 4049:2009.
8. Watts, D. C.; Marouf, A. S.; Al-Hindi, A. M. *Dent. Mater.* **2003**, *19*, 1-11.
